# Viral infection switches the balance between bacterial and eukaryotic recyclers of organic matter during coccolithophore blooms

**DOI:** 10.1038/s41467-023-36049-3

**Published:** 2023-01-31

**Authors:** Flora Vincent, Matti Gralka, Guy Schleyer, Daniella Schatz, Miguel Cabrera-Brufau, Constanze Kuhlisch, Andreas Sichert, Silvia Vidal-Melgosa, Kyle Mayers, Noa Barak-Gavish, J. Michel Flores, Marta Masdeu-Navarro, Jorun Karin Egge, Aud Larsen, Jan-Hendrik Hehemann, Celia Marrasé, Rafel Simó, Otto X. Cordero, Assaf Vardi

**Affiliations:** 1 Department of Plant and Environmental Sciences, Weizmann Institute of Science, 7610001 Rehovot, Israel; 2 Department of Civil and Environmental Engineering, Massachusetts Institute of Technology, Cambridge, 02145 MA USA; 3 Institut de Ciències del Mar, CSIC, 08003 Barcelona, Spain; 4 Max Planck Institute for Marine Microbiology, 28359 Bremen, Germany; 5 Center for Marine Environmental Sciences (MARUM), University of Bremen, 28359 Bremen, Germany; 6 NORCE Norwegian Research Centre, 5008 Bergen, Norway; 7 Department of Earth and Planetary Science, Weizmann Institute of Science, 7610001 Rehovot, Israel; 8 Department of Biological Sciences (BIO), University of Bergen, 5020 Bergen, Norway; 9 Present Address: Developmental Biology Unit, European Molecular Biological Laboratory, 69117 Heidelberg, Germany; 10 Present Address: Systems Biology Lab, Amsterdam Institute for Life and Environment (A-Life)/Amsterdam Institute of Molecular and Life Sciences (AIMMS), Vrije Universiteit Amsterdam, 1081 Amsterdam, The Netherlands

**Keywords:** Microbial ecology, Marine biology, Water microbiology, Virus-host interactions

## Abstract

Algal blooms are hotspots of marine primary production and play central roles in microbial ecology and global elemental cycling. Upon demise of the bloom, organic carbon is partly respired and partly transferred to either higher trophic levels, bacterial biomass production or sinking. Viral infection can lead to bloom termination, but its impact on the fate of carbon remains largely unquantified. Here, we characterize the interplay between viral infection and the composition of a bloom-associated microbiome and consequently the evolving biogeochemical landscape, by conducting a large-scale mesocosm experiment where we monitor seven induced coccolithophore blooms. The blooms show different degrees of viral infection and reveal that only high levels of viral infection are followed by significant shifts in the composition of free-living bacterial and eukaryotic assemblages. Intriguingly, upon viral infection the biomass of eukaryotic heterotrophs (thraustochytrids) rivals that of bacteria as potential recyclers of organic matter. By combining modeling and quantification of active viral infection at a single-cell resolution, we estimate that viral infection causes a 2–4 fold increase in per-cell rates of extracellular carbon release in the form of acidic polysaccharides and particulate inorganic carbon, two major contributors to carbon sinking into the deep ocean. These results reveal the impact of viral infection on the fate of carbon through microbial recyclers of organic matter in large-scale coccolithophore blooms.

## Abstract (web summary)

Algal blooms are hotspots of marine primary production that play central roles in microbial ecology and global elemental cycling. Here, the authors show how bloom termination by viral infection can shift the balance between eukaryotic and prokaryotic recyclers of phytoplankton biomass.

## Introduction

Marine algae are responsible for half of Earth’s primary production and form the basis of the oceanic food chain^1^. Algal blooms^2^ are ephemeral events of phytoplankton proliferation that occur annually across the globe^3^ covering thousands of square kilometers. Upon bloom demise, most of the fixed carbon is transferred to higher trophic levels either via herbivorous predation or through heterotrophic bacteria and their predators (a process called the “microbial loop”), being largely recycled and respired along the way^4,5^. Only a minor fraction of the algal biomass is sequestered into the deep sea. It has long been hypothesized that the cause of bloom termination affects the associated microbiome and fate of carbon. Viral infection enhances lysis of host cells and release of dissolved organic matter (DOM), leading to bacterial growth and respiration at the expense of organic carbon sinking, in a process coined the “viral shunt“^6^. It has also been suggested that viral infection increases particle formation and thus biomass sinking. Thus, infection could accelerate the biologically driven sequestration of carbon into the deep sea in the so-called “viral shuttle” process^7,8^. However, we still lack quantitative assessment of how viruses alter microbial composition and influence the fate of carbon during algal blooms.

The unpredictability of oceanic blooms makes it challenging to monitor their microbial succession at high temporal resolution. Mesocosm experiments are therefore an important experimental setup in plankton ecology^9^ that mimic as closely as possible the complexity of marine microbial ecosystems. Here, in order to provide a quantitative view of viral infection and its effect on carbon flow in the ocean, we performed a mesocosm experiment to investigate the bloom dynamics of the cosmopolitan calcifying microalga *Emiliania huxleyi* in seven large enclosures, anticipating spontaneous emergence of viral infection^10^. The enclosures were immersed in a fjord near Bergen, Norway, filled with 11 thousand liters of fjord water containing natural planktonic communities and nutrients were added on a series of consecutive days. Each enclosure spontaneously showed absent, moderate, or high levels of viral infection of *E. huxleyi* by its large double stranded DNA virus EhV^11^. Combining daily monitoring of a variety of biological and biogeochemical parameters, we quantified the impact of viral infection on the surrounding eukaryotic and bacterial communities and on carbon cycling from the cellular to the biogeochemical level. Our results demonstrate how viruses impact microbial communities in coccolithophore blooms and their biogeochemical consequence on the fate of carbon.

## Results

### Succession of prominent community members during algal bloom dynamics

**Fig. 1:**
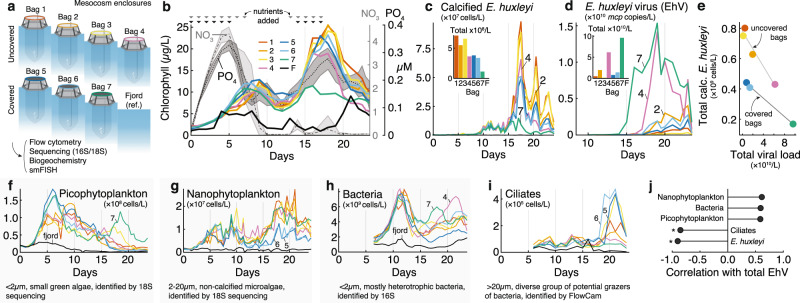
An overview of the mesocosm setup and dynamics of prominent community members. **a** Schematic view of the seven mesocosm enclosures during the mesocosm experiment. Unfiltered fjord water was used as the microbial inoculum seeding the enclosures and was sampled as a reference for microbial dynamics under natural conditions. Bags 1–4 were open, bags 5–7 were fitted with an airtight cover to measure aerosols. **b** Fluorometric chlorophyll measurements (left axis), where each color corresponds to a different bag and the fjord in black (F). Nitrate and phosphate concentrations over time averaged across enclosures (right axes) and the shaded value represents standard deviation. Arrows on the top indicate nutrient addition with nitrogen in gray and phosphorus in black. **c** Calcified *E. huxleyi* abundance measured by flow cytometry, based on high side scatter and high chlorophyll signals. The small bar chart shows the integrated abundance of *E. huxleyi* over time (see Methods). **d** Concentration of EhV based on qPCR of *mcp* (major capsid protein) gene in 2–20 μm pore filters. The small bar chart shows the integrated abundance of EhV over time. Hereafter, we refer to EhV as total viral load for simplicity. **e** Scatter plot of total calcified *E. huxleyi* abundance as a function of total viral abundance, with a linear model fit for covered and uncovered bags. **f–i** Absolute abundances of key players in the microbial succession, sorted by peak abundance time: **f** picophytoplankton abundance measured by flow cytometry, based on low side scatter and low chlorophyll signals; **g** non-calcifying *E. huxleyi* and other nanophytoplankton abundance measured by flow cytometry, based on low side scatter and high chlorophyll signals; **h** Absolute abundance of bacteria measured by flow cytometry after SYBR green staining; **i** Ciliate abundance measured by imaging flow microscopy and annotated using EcoTaxa. **j** Correlation between EhV viral load and average planktonic abundances (corrected for bag cover) across bags. Asterisks (*) indicate significant correlations (linear model, *p* < 0.01). Source data are provided as a Source Data file.

Our mesocosm experiment consisted of four uncovered enclosures (bags 1–4) and three air-tight sealed enclosures to collect aerosols (bags 5–7) (Fig. 1a). For 24 days, we monitored phytoplankton and bacterial cell counts using flow cytometry, determined microbiome composition using metabarcoding (bacterial and eukaryotic), measured various biogeochemical parameters (see Methods), and determined the level of viral infection in single cells using single molecule fluorescent in situ hybridization (smFISH)^12^. These biological and chemical features were compared to the surrounding fjord waters used as a control for microbial dynamics under natural conditions.

In the initial phase (Day 0–10), the enclosures were nitrate and phosphate replete following nutrient addition; at later stages (Day 10–23), the enclosures were nitrate, but not phosphate, limited (Fig. 1b)*.* Bulk chlorophyll measurements displayed two peaks in all enclosures, with a first smaller phytoplankton bloom reaching 12.5 μg/L of chlorophyll (Day 0–10) followed by a second bloom reaching 25 μg/L of chlorophyll (Day 10–23) (Fig. 1b). Calcified *E. huxleyi* cells, quantified by flow cytometry and identified by scanning electron microscopy, dominated the second bloom and were noticeable from the milky color of the water due to the algae’s calcium carbonate shell (Fig. 1c, Supplementary Fig. 1). *E. huxleyi* is a cosmopolitan bloom-forming alga and a major calcite producer, causing the transport of large amounts of carbon into the ocean’s sediments^13^. In addition to lower average *E. huxleyi* cell abundance in covered relative to uncovered enclosures, we also observed stark differences in *E. huxleyi* demise dynamics across bags (Day 18–24), with up to 90% lower algae abundances in bags 4 and 7 compared to the other bags. Previous studies attribute the demise of natural blooms of *E. huxleyi* mainly to virus-associated mortality caused by the *E. huxleyi*-specific large coccolithovirus (EhV)^14–16^, however other environmental conditions can limit their growth (e.g., lack of nutrients). To estimate the relative importance of viral demise in our mesocosms, we quantified the abundance of EhV in the free-living and particle-attached size fractions using flow-cytometry and qPCR of the major capsid protein (*mcp*) gene (see Supplementary Fig. 2 for comparisons). In the 2–20 μm size fraction (to focus on infected *E. huxleyi* cells and viral particles associated with its biomass), viral abundance varied considerably between enclosures (Fig. 1d). Bag 7 (covered) and bag 4 (uncovered) showed high concentrations of biomass-associated EhV with up to 1.54 × 10^10^ *mcp* copies/L and 1.42 × 10^10^ *mcp* copies/L, respectively, while bag 5 (covered) and bag 3 (uncovered) showed viral loads three orders of magnitude lower. Viral abundance had a direct negative correlation with algal abundance, in which viral concentrations explained 92% of the variance in *E. huxleyi* density across bags 1–4 and 99% of the variance in bags 7–9, suggesting that viruses control the magnitude of an *E. huxleyi* bloom (Fig. 1e). Note, however, that bloom demise was observed even with low or no viral infection (e.g., bags 1 and 3) suggesting that other mortality agents are important in *E. huxleyi* blooms. For instance, *E. huxleyi* abundances started to decrease in all enclosures after nutrient addition was ceased on day 17, and nitrate remained depleted until the end of the experiment, suggesting potential nutrient limitation as a contributor to *E. huxleyi* demise in our experiment. In addition, in enclosures with low viral load (bags 1, 3, 5, and 6), we observed up to a sixfold increase in ciliates (measured by imaging flow microscopy, Fig. 1i) that could potentially graze on *E. huxleyi*^17^.

The first phytoplankton bloom (Day 0–10) which we termed the mixed bloom, preceding the *E. huxleyi* bloom, was dominated by the pico-phytoplankton *Bathycoccus*, *Micromonas*, and *Leptocylindrus minimus* as well as small dinoflagellates, representing over 80% of the community in the 0.2–2 μm size-fraction (Supplementary Fig. 3). This bloom reached 1.81 × 10^8^ cells/L in bag 5 (Fig. 1f). Nano-phytoplankton (Fig. 1g) were also important players in this mixed bloom and sequencing of the 2–20 μm size fraction 18S rDNA revealed that dinoflagellates (Group-I Clade-I) were especially abundant (see further information below).

Phytoplankton cells fix inorganic carbon into organic biomass, and part of it can be secreted in the form of metabolites that heterotrophic bacteria can utilize for their growth^18–21^. Interestingly, the dissolved organic carbon (DOC) concentration increased only moderately after each of the blooms (Supplementary Fig. 4). This could be explained by a fast-bacterial assimilation as we observed a more than tenfold exponential increase in bacterial abundance between days 5 and 13 (Fig. 1h), doubling every 24–36 h. By contrast, bacteria were less abundant during the *E. huxleyi* bloom and demise compared to the mixed bloom, showing an average of less than twofold increase after day 20. In the two most infected bags, bag 4 and bag 7, the increase in bacterial abundance was two to three-fold during the demise phase. Overall, total viral load in the different enclosures was significantly negatively correlated with the abundance of host (*E. huxleyi*) and grazer (ciliates) concentrations but not with pico-nano-phytoplankton or bacteria abundances (Fig. 1j, Supplementary Fig. 5). The negative correlation between grazing and viral lysis was supported via grazing dilution assays across different mesocosms (Supplementary Fig. 6), suggesting that these two top-down mortality agents competed during algal blooms.

### The impact of viral infection on the composition of microbial assemblages

**Fig. 2:**
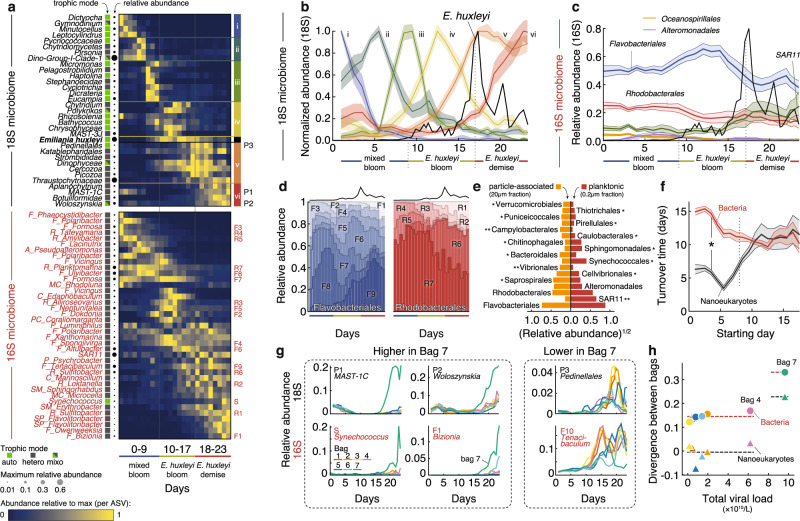
Microbial succession during the growth and demise of algal blooms with different viral loads. **a** Nanoeukaryotic (black) and bacterial (red) microbial succession throughout the experiment duration, averaged across enclosures. Each row is a taxon with bacteria (ASVs) in red and eukaryotes (named genera) in black. The trophic modes of each ASV are detailed in the box color with autotrophs in green, heterotrophs in gray, and mixotrophs in green/gray. Days are shown in columns. 18S species are grouped by clusters of different colors and taxa of special interest (**d**, **g**) are indicated on the right of the heatmap. 16S abbreviations denote taxonomy at the order level: F: Flavobacteriales, R: Rhodobacterales, A: Alteromonadales, C: Cytophagales, CV: Cellvibrionales, MC: Micrococcales, P: Pseudomonadales, PC: Puniceicoccales, SM: Sphingomonadales, SP: Saprospirales. **b** Succession of 18S-based ASVs in the 2–20 μm fraction, clustered by similarity of their relative abundance dynamics averaged across bags. The shaded area represents the standard deviation within each cluster, centered around the mean normalized abundance of species in that cluster. The absolute abundance of *E. huxleyi* enumerated with flow cytometry is overlaid as a guide (black line, not to scale). Each cluster is normalized to its own maximum abundance and their species composition is detailed in (**a**, **c**). Relative abundance of major bacterial orders throughout the bloom, averaged for all enclosures using 16S amplicon sequencing of the 0.2–2 μm fraction. The absolute abundance of *E. huxleyi* enumerated with flow cytometry is overlaid as a guide (black line, not to scale). Shaded areas represent standard deviations across bags. **d** Relative abundance of different Flavobacteriales and Rhodobacterales genera within each order averaged across all enclosures. The dark line on the top represents *E. huxleyi* abundance trends as a guide. The letters F1-F10 and R1-R7 refer to (**a**). **e** Relative abundance of bacteria in the free-living versus particle-attached size fractions. Asterisks indicate fold-differences between size fractions (*: >2.5; **: >10). **f** Rate at which bacteria and nanoeukaryotic community similarities change over time. Nanoeukaryotic communities initially turnover much faster than the bacterial ones (for each time point until day 8, *p* < 0.01 by two-sided Mann–Whitney test with Bonferroni multiple testing correction). Lines and shaded areas represent mean and standard deviation across bags, respectively. **g** Selection of eukaryotic and bacterial ASVs that are overrepresented or underrepresented in bag 7. The letters P1, P2, P3 and S, F1, F10 refer to (**a**). **h** Correlation, per bag and per 16S or 18S ASV, between total viral load and percentage dissimilarity in microbial composition from one day to another. The divergence of a bag is defined as the change in pairwise Bray–Curtis distance between the focal bag and all other bags from the start of the *E. huxleyi* bloom to its demise. Source data are provided as a Source Data file.

To understand how eukaryotic viral infection can alter planktonic community composition, we conducted microbiome profiling of all the mesocosm enclosures. We opted for a detailed time series of the 16S rRNA amplicons based on samples collected daily of both the planktonic (0.2–2 μm) and particle-associated bacterial (20–200 μm) size fractions. The 2–20 μm size fraction was ignored due to contamination of 16S from *E. huxleyi* chloroplasts. These measurements were coupled with assessment of the nanoeukaryotic diversity by 18S  rRNA amplicon sequencing of the 2–20 μm size fraction communities. Throughout the two blooms, we observed a repeatable pattern of eukaryotic and bacterial taxa successions (Fig. 2a). The relative abundance of *E. huxleyi* can be used to define three major phases: the mixed bloom, (days 0–9), the exponential growth phase of *E. huxleyi* (days 10–17), and its demise (days 18–23). Nanoeukaryotes, clustered according to the relative abundance patterns at the genus level, showed a rapid succession of boom-and-bust cycles, each about 5–10 days long (Fig. 2b, Supplementary Figs. 7, 8). Unique clusters of nanoeukaryote species bloom upon *E. huxleyi* growth (Cluster V) and demise (Cluster VI), thus defining a bloom associated protist microbiome.

By comparison, bacterial succession was much less dynamic in the free-living community: the composition at the order level was relatively stable (Fig. 2c). The mixed bloom was associated with bacterial groups known to be involved in algal biomass remineralization, such as Flavobacteriales^22,23^ and Rhodobacterales. Throughout the *E. huxleyi* bloom, we observed a slow, more than ten-fold increase in the relative abundance of SAR11, a cosmopolitan bacterial clade in the oligotrophic ocean^24^. This apparent facilitation of SAR11 growth by *E. huxleyi* is in line with previous observations of their co-occurrence^25^ and could be mediated by the organosulfur compound dimethylsulfoniopropionate (DMSP), which *E. huxleyi* produces and excretes^26,27^, and that SAR11 can utilize as a source of reduced sulfur^28^. In contrast to the relative stability of the bacterial composition at the order level, there were clear successions at the genus level within the two dominant bacterial orders, Flavobacteriales and Rhodobacterales (Fig. 2d, Supplementary Fig. 9). The *E. huxleyi* bloom and demise coincided with the relative increase of two genera: *Tenacibaculum*, a potential fish parasite frequently associated with algal blooms^23^, and *Sulfitobacter*, a genus containing DMSP degrading species that are pathogenic to *E. huxleyi* cells^27^. Gammaproteobacteria such as *Vibrionale*s, *Cellvibrionales*, and, *Alteromonadales*, often reported as dominant members in bloom-associated communities^29^, remained at low relative abundance (<10% of the reads at its maximum) in the planktonic populations. However, analysis of the particle-associated microbiome on a subset of bags (limited to bags 2, 3, 4, 7 of the >20 μm), showed enrichment of *Vibrionale*s in the larger fraction as well as other clades such as *Campylobacterales* and *Saprospirales*, that were rare in the pico-planktonic phase (Fig. 2e, Supplementary Fig. 10). However, Flavobacteriales and Rhodobacterales remained the dominant orders in the particle-attached fraction. Interestingly, the concentration of bacteria and their growth rate in this larger size fraction was much smaller than in the free-living size fraction (Supplementary Figs. 11, 12).

Using this data, we can potentially distinguish two different types of microbiome changes specifically associated with the demise of each bloom. Protist species that increased during the mixed bloom demise (Fig. 2b, cluster IV) include heterotrophs such as *Chytridium* (potential parasites), *Polykrikos* (dinoflagellate predator), *MAST-3J* (bacterivorous nanoflagellates) but also photosynthetic chrysophytes, albeit in lower abundance, while different heterotrophs clearly dominated the *E. huxleyi* demise (see below). On the bacterial side it does seem that *Neptunitalea* (F5 in Fig. 2a and Fig. 2d), *Formosa* (F7), *Alliroseovarius* (R3) were specific to the demise of the mixed bloom, while *Biziona* (F1), *Sulfitobacter* (R1, R6) and *Loktanella* (R2) seem to be more associated with the *E. huxleyi* bloom demise. To further compare and contrast bacterial and eukaryotic dynamics, we computed their turnover time, defined by the exponential rate at which the Bray-Curtis similarity declined over time (see Methods). Given their small size and known fast growth rates, we expected heterotrophic bacteria to respond much faster to our nutrient additions (N and P) than eukaryotes. However, eukaryotes were the first responders to nutrient addition, and their assemblage turned over much faster (every 5 days initially) than bacteria, which only showed significant growth toward the end of the first bloom (turnover every 10 days) (Fig. 2f). The sequence of response to the nutrient addition can be explained by the direction of nutrient flow in phytoplankton blooms when nutrients increase: eukaryotes, especially phytoplankton, were likely nitrogen and/or phosphorous-limited at the start of the experiment, whereas bacteria appeared to be carbon-limited and required organic carbon released upon demise of the first mixed bloom in order to grow.

Despite strong compositional similarities of clusters amongst the seven enclosures, the bacterial and nanoeukaryotic assemblages gradually diverged between enclosures after the mixed bloom. During the *E. huxleyi* bloom demise, bag 7 (the most virally infected) diverged in microbiome composition from the other enclosures (Supplementary Fig. 13). Eukaryotes such as MAST-1C (a heterotrophic flagellate), *Woloszynskia* (a mixotrophic dinoflagellate), as well as the cyanobacterium *Synechococcus* and the bacterium *Bizionia* (family *Flavobacteriaeceae*) were overrepresented in bag 7 (Fig. 2g, Supplementary Figs. 14, 15). The growth of *Synechococcus* during high viral infection suggests that the resulting flux of DOM benefits not only heterotrophic but also autotrophic bacterial growth, both in the free-living and particle-associated fractions^30,31^. Recent ecosystem modeling suggests this may be due to efficient recycling of growth-limiting nutrients in the photic zone during viral infection^32^. The eukaryotes *Pedinellales* (autotroph) and the bacterium *Tenacibaculum* (family *Flavobacteriaeceae*) grew less in bag 7 than in the rest of the enclosures. In contrast to these observations in the highly infected bag 7, the moderately infected bag 4 showed a 16S and 18S-based composition that did not diverge from the less infected enclosures (Fig. 2h). These findings suggest that substantial change in microbial assemblages during virus-associated *E. huxleyi* demise in our experiment was conditional on high viral infection levels.

### Viral infection impacts the composition of organic matter recyclers

During *E. huxleyi* demise, a large pool of dissolved organic carbon (DOC) derived from lysed phytoplankton biomass became available for bacterial recycling, with estimates of about 270 μg C/L/day from *E. huxleyi* alone (see Methods). Yet, the growth of both free-living bacteria (quantified by flow-cytometry) and particle-attached bacteria was moderate (Supplementary Figs. 11, 12). This could be explained by several factors, including the removal of bacteria by aggregation and sinking, or increased bacterial cell death by phages or bacterivores^33^. However, the abundance of typical bacterivores like dinoflagellates remained low and ciliate abundance only increased late into the demise phase of the *E. huxleyi* bloom (day 20–23) (Fig. 1i). The low number of predators, combined with the observation that DOC concentration stabilized during bloom demise, led us to hypothesize that bacteria competed for nutrients with another group of organic matter recyclers.

**Fig. 3:**
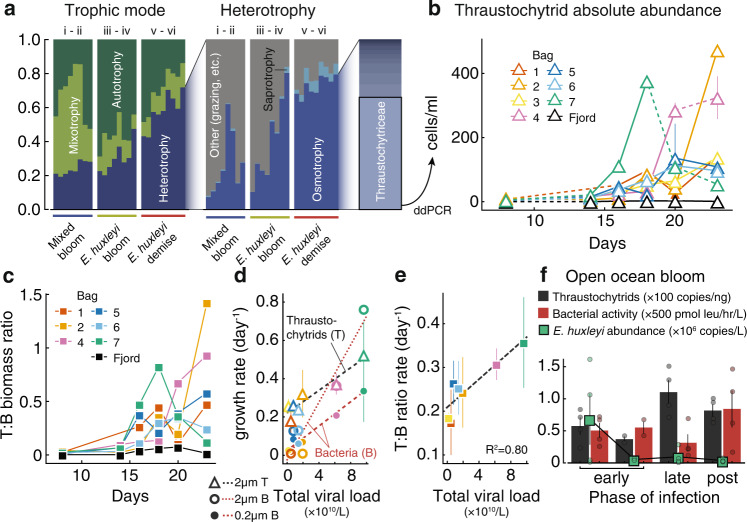
Viral induced bloom demise changes the composition of organic matter recyclers. **a** Analysis of predicted eukaryotic traits plotted per phase of the *E. huxleyi* bloom. Relative abundance of autotrophy, mixotrophy, and heterotrophy, defined through literature search (left). Trophic modes within heterotrophy, as defined by^72^ (right). **b** Absolute abundance of thraustochytrids throughout the mesocosm experiment on the 2–20 μm size fraction, measured by ddPCR. Points and error bars show the mean and standard deviation across *n* = 3 replicates. **c** Thraustochytrid to bacteria biomass ratios throughout the experiment duration (see Methods and Supplementary Data 1–4 for detailed calculation). **d** Correlation between total viral load and growth rate during demise phase of thraustochytrids (2–20 μm) and bacteria (for the free-living fraction measured by flow-cytometry and for 2–20 μm fraction measured by qPCR). Symbols indicate the mean, error bars the standard deviation between parameters obtained from *n* = 3 fits with shifted (by ±0.5 days) start times. **e** Exponential rate of change of the biomass ratio of thraustochytrids to bacteria (combining biomasses from free-living and 2–20 μm size fraction) plotted as a function of total viral load, per bag (see Methods). Symbols and error bars represent the mean and standard error for parameter estimates of the nonlinear fits in (**c**). **f** Concentration of thraustochytrids measured by ddPCR, *E. huxleyi* cells measured by qPCR, and bacterial production using leucine incorporation^46^, in three phases of an open ocean *E. huxleyi* bloom infection. Transparent symbols are individual measurements; bars, solid, and error bars represent means and standard error of *n* = 4 samples (*n* = 2 for second “early” phase of infection). Source data are provided as a Source Data file.

To identify other heterotrophs, we re-examined the eukaryotic microbiome in search for organic matter recyclers. Functional annotation of the nanoeukaryotes (see Methods) revealed that while eukaryotic assemblages were composed of autotrophs and mixotrophs during the first mixed bloom, heterotrophs, and specifically osmotrophs, became highly abundant through the *E. huxleyi* bloom and demise (Fig. 3a). These heterotrophs were dominated by thraustochytrids (*Thraustochytriaceae* in Fig. 2a), members of a diverse lineage of eukaryotic osmotrophs^34^, which contributed over 50% of all 18S rDNA reads in the 2–20 μm size fraction during bloom demise, across all bags. Thraustochytrids are known to possess an arsenal of extracellular digestive enzymes, making them important decomposers of organic matter in coastal sediments^35^ and deep-sea particles^36^. With their large intracellular lipid reserves, they also serve as an important food source for higher trophic levels^37^. However, the importance of thraustochytrids in microbial food webs has yet to be explored. During algal blooms, they could potentially play a significant role as decomposers^38^, bacterivores, or even parasites^39^. Some members of the group are also known to produce ectoplasmic nets, through which they can extract intracellular nutrients of preyed cells^40,41^ such as senescent diatoms^42,43^.

In order to quantify the absolute abundance of thraustochytrids, we performed digital droplet PCR (ddPCR) targeting thraustochytrid 18S rDNA across all mesocosm enclosures on the 2–20 μm and 20–200 μm filters. While undetected during the mixed bloom, thraustochytrids cell abundance (estimated based on values of 64 18S rDNA copies/cell, see Methods) reached over 400 cells/ml in the 2–20 μm size fraction (Fig. 3b) and was negligible in the 20–200 μm size fraction (we thus ignore thraustochytrid measurements from the 20–200 μm). Thraustochytrids’ total biomass (using a conversion factor of 1.65 × 10^−10^ g of carbon/cell^44^) increased steadily after day 16 and was comparable to that of bacteria (combining both free-living bacterial abundance quantified by flow-cytometry and particle-attached bacterial abundance estimated by qPCR) during the *E. huxleyi* bloom demise (Fig. 3c, Supplementary Figs. 16, 17, Supplementary Data 1–4). Thus, *E. huxleyi* demise reproducibly triggered the biomass increase of eukaryotic and bacterial degraders in all bags.

In order to examine if viral associated demise impacted thraustochytrid growth, we compute their growth rate compared to that of bacteria as a function of viral load. Thraustochytrids grew particularly faster in the bags with high viral load (Fig. 3d), similarly to bacteria from both the free-living and 2–20 μm (we ignored bacteria from the 20–200 μm due to their low concentration). Thus, viral-associated *E. huxleyi* demise enhanced the growth of both eukaryotic and bacterial degraders, consistent with the “viral shunt” hypothesis, and bacteria on the 2–20 μm displayed the highest growth rate. In terms of biomass, however, viral load was well correlated (*R*^2^ = 0.80) with the exponential rate at which the ratio of thraustochytrid to bacterial biomass increased (Fig. 3e). This means that while bacteria grew faster, thraustochytrids biomass increased faster than bacterial biomass in bags with strong viral infection of *E. huxleyi*. To test whether this was also true in oceanic blooms, we examined samples collected from an open-ocean *E. huxleyi* bloom in the North Atlantic where different phases of viral infection—early or late—were derived from lipid markers and viral transcripts^45^. Thraustochytrid abundances were quantified by ddPCR. Since no direct data on the absolute abundance of bacteria was collected during this cruise, we used previously measured bacterial production rates of the same samples^46^, which typically correlate with bacterial growth^47^. In line with our mesocosm results, we detected higher thraustochytrid abundance and lower bacterial production during later stages of viral infection relative to the early phase of bloom infection (Fig. 3f), suggesting that thraustochytrids also benefitted from viral-associated *E. huxleyi* bloom demise. Sequencing of larger 18S rRNA fragments from the mesocosm and open ocean samples revealed a single dominant species across these ecosystems, whose closest relative is an uncultivated clone (94% identity). Taken together, these results suggest that this thraustochytrid species potentially specializes on exudates from *E. huxleyi* viral demise (Supplementary Fig. 18).

### Viral infection enhances population-level and per-cell rates of carbon release

During *E. huxleyi* blooms, which can cover over 100,000 square kilometers in the ocean^48^, cell concentrations of this species can account for 75% or more of the total abundance of photosynthetic plankton in the area^48^. The algal biomass and the coccoliths that form the *E. huxleyi’s* calcified shell have a profound impact on the carbon cycle and global CaCO_3_ export flux^49^. We therefore investigated the biogeochemical consequences of viral infection of *E. huxleyi* blooms by quantifying two extracellular components of the carbon cycle: the organic carbon in the form of transparent exopolymer particles (TEP), and the particulate inorganic carbon (PIC) that results from calcification.

**Fig. 4:**
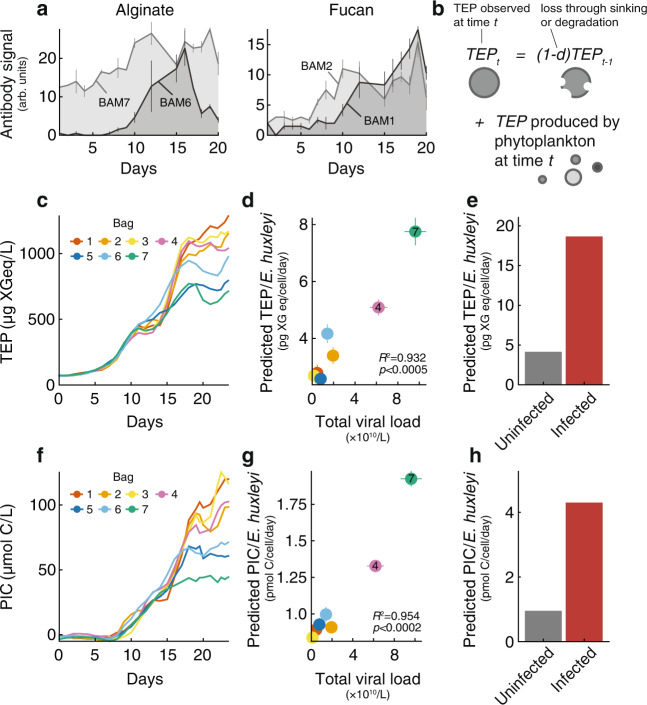
Viral infection promotes release of PIC and TEP production from a coccolithophore bloom. **a** Alginate and fucan abundance in particulate organic matter (POM) over time, based on mixed water from bags 1–4, measured by carbohydrate microarray analysis. BAM1, 2, 6 and 7 correspond to glycan-specific monoclonal antibodies, used to measure the relative abundance of their recognized polysaccharide epitopes in POM water extracts. Lines correspond to mean of between *n* = 2 and *n* = 5 filters used per day, error bars represent standard deviations across samples. **b** Scheme of TEP modeling, as a function of phytoplankton concentrations and degradation rate which enables prediction of the *E. huxleyi* contribution to the TEP pool. **c** TEP concentration measured by Alcian blue staining over time, per bag. **d** Predicted TEP/cell as a function of total viral load for each bag, for *E. huxleyi* cells. **e** Predicted TEP/cell secretion in infected versus non-infected *E. huxleyi* cells using intracellular measurements of actively infected single cells. **f** PIC production during algal bloom succession. **g** Predicted PIC/cell as a function of total viral load for each bag, for *E. huxleyi* cells. **h** Predicted PIC/cell production in infected versus non-infected *E. huxleyi* cells using intracellular measurements of actively infected single cells. Symbols and error bars in (**d** and **g**) represent mean and standard error of the parameter estimate from the model. Source data are provided as a Source Data file.

TEP are made of acidic polysaccharides that form due to abiotic coagulation of dissolved carbohydrates secreted by phytoplankton. TEP are an important component of the marine particulate organic carbon, and represent a potential source of food for bacteria or other heterotrophs^50,51^. Yet, recent work suggests that some polysaccharides within TEP can be recalcitrant for microbes, hampering its degradation^52^. TEP are an essential vector for carbon export by triggering cell aggregation and sinking, but their chemical composition has only recently been elucidated. To better identify the polysaccharides in TEP during *E. huxleyi* blooms, we used carbohydrate microarray analysis on the particulate fraction^53^. Out of the alginate and sulfated fucans epitopes, the ones recognized by the monoclonal antibodies BAM6^54^ and BAM1^55^ respectively, accumulated during the *E. huxleyi* bloom and are thus likely *E. huxleyi*-related (Fig. 4a). BAM6 signal decreased during the demise phase, suggesting potential degradation of its recognized epitope by the demise-associated microbiome. In contrast, the accumulation of the epitope detected with BAM1 suggests that this sulfated fucan did not serve as a substrate for thraustochytrids or bacteria^56^, but may be part of TEP and thus relevant for carbon export via sinking particles^52^.

To decipher the effects of viral infection on TEP production, we modeled TEP concentration as a function of its producers’ abundances (*E. huxleyi*, picophytoplankton, and non-calcified nanophytoplankton such as diatoms, dinoflagellates and haptophytes defined by flow-cytometry) and a TEP loss rate through sinking or degradation (Fig. 4b) that we fitted to in situ TEP measurements (Fig. 4c) (see Methods). The model described the TEP abundance well, achieving an average ${R}^{2}$ of 0.988 to observations. Using the model, we estimated that the amount of TEP produced per *E. huxleyi* cell per day was 60–75% of the total TEP pool at the onset of bloom demise (Supplementary Fig. 19). There was a strong dependence of estimated TEP per cell on viral infection: TEP production per *E. huxleyi* cell was more than twice as high in the infected bag 7 than in non-infected bags, which was not the case for other phytoplankton groups (Supplementary Fig. 20). Across all bags, there was a strong correlation to total viral load (*R*^2^ = 0.932, *p* < 0.0005, Fig. 4d), consistent with previous results suggesting higher export during viral-associated *E. huxleyi* blooms in open ocean and mesocosm experiments^10,45^. This suggests that, at the population level, *E. huxleyi* cells secreted twice the amount of organic carbon in presence of high viral load. To validate this correlation, we applied the same model for particulate organic carbon (POC) production. The model gave an excellent fit to observations (${R}^{2}$ > 0.98 across all bags) but the estimate for the amount of organic carbon per *E. huxleyi* cell (4–6 pg C/cell, in line with other estimates^57^ (Supplementary Fig. 21) was uncorrelated with the total viral load (*p* > 0.05).

Since viral infection remodels the algal host metabolism^58,59^, we hypothesized that infected and non-infected cells in the same bloom may differ in their actual TEP production, and sought to quantify this process as opposed to simply averaging TEP over the entire bulk population. To differentiate infected from non-infected cells, we used smFISH to probe viral mRNA in single *E. huxleyi* cells^12^ and obtained a time-course of the fraction of actively infected cells in two different enclosures (Supplementary Fig. 22). At most, 10 and 25% of all *E. huxleyi* cells were infected in bags 2 and 4 respectively, reflecting the heterogeneity of cell fates within each bloom succession and demise and providing us with a gradient of intracellular viral infection dynamics. By assuming that non-infected cells produced the same amount of TEP regardless of the bag’s viral load, we estimated that an infected *E. huxleyi* cell produced ~19 pg xanthan gum (XG) equivalent/day (see Methods), or 4 times more TEP than its non-infected bystander cell (Fig. 4e). Notably, viral infection did not increase secretion of proteinaceous material: the measurement and modeling of protein-rich particles (Coomassie Stained Particles) (Supplementary Fig. 23) showed no correlation with viral load, indicating that the cellular response to infection is specific to some metabolic products.

Particulate inorganic carbon (PIC) in the form of calcium carbonate is the basis of one of the main processes making up the marine carbon cycle, the carbonate pump, by which inorganic carbon is exported along with organic matter to the deep ocean. A major part of PIC in the ocean is comprised of coccolithophore shells, particularly *E. huxleyi’s* coccoliths^60^. PIC accumulated over time in our study (Fig. 4f). We fitted the PIC curves to a model accounting for *E. huxleyi* coccolith production, a degradation rate, and a term allowing for shedding and re-calcification (see Methods). Like TEP, predicted PIC per *E. huxleyi* cell at the population level was significantly correlated to total viral load from about 1 to 2 pmol PIC/cell/day (${R}^{2}=0.954,\,p \, < \,0.0002,$ Fig. 4g), which is consistent with lab-based measurements^61^. Using the measured fraction of active single-cell infection, we estimated that infected single cells produced 4 times more PIC per cell than their non-infected bystander cells (Fig. 4h). Overall these data suggest that active viral infection can have remarkable consequences on exportable carbon (TEP and PIC) release both on the population-level (twofold increase) and per infected cell (fourfold increase).

## Discussion

**Fig. 5:**
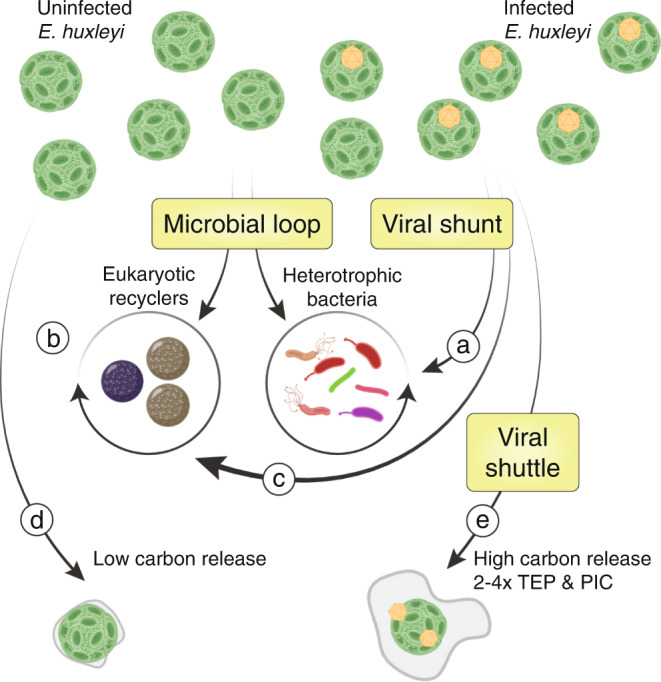
Consequences of viral infection on microbial community composition and carbon cycling. **a** The bacterial and eukaryotic microbiomes are remodeled in response to viral infection only when level of infection is high. **b** Thraustochytrid rival bacteria as significant recyclers of organic matter during *E. huxleyi* demise. **c** Thraustochytrids benefit from viral infection of *E. huxleyi*. **d** When the demise is not virus-associated, *E. huxleyi* cells release a small amount of organic and inorganic carbon. **e** Viral infection increases *E. huxleyi* cells carbon release between 2 and 4-fold under the form of TEP and PIC as compared to (**d**). Arrows represent the direction of carbon flow. Created with BioRender.com.

Here we provided in-depth characterization of the microbial and biogeochemical dynamics of two successive algal blooms in seven mesocosm enclosures, which provides a unique experimental platform to quantify the consequences of viral infection at the ecosystem level in high temporal resolution. Starting from the same microbial inoculum, our mesocosm enclosures underwent ordered microbial successions that culminated in blooms^11,62^ of the coccolithophore *E. huxleyi*. Despite identical starting conditions, the blooms presented varying degrees of viral infection and concomitant changes in the surrounding microbiome and biogeochemical cycling. We used this spontaneous emergence of differential bloom and demise dynamics to make three critical observations regarding the microbial ecology and the biogeochemical effects of algal blooms and their viral infection (Fig. 5), generating novel hypotheses for future lab-based mechanistic studies.

First, we showed that major changes in the free-living bacteria and nanoeukaryotes microbiomes are only observed when there is a high level of viral infection (Fig. 5a). Given that only one in seven enclosures experienced such high levels of viral lysis, its occurrence in natural ecosystems may be rare but can, along with other processes like protozoan grazing, phage infection, and possible interactions with pathogenic bacteria, profoundly impact microbial diversity and community composition. It is also possible that the microbiome response takes longer than the duration of our experiment or may be localized to *E. huxleyi*-attached communities. However, if viral infection enhances TEP production, our results suggest that the large particle-associated microbiome is overall quite similar to the free-living one, except for a few bacterial orders that are specifically particle-associated but that represent a small proportion of the total bacterial abundance.

Second, we estimated that the biomass of eukaryotic osmotrophs can be comparable to that of heterotrophic bacteria during *E. huxleyi* blooms and demise (Fig. 5b) and in particular during viral infection (Fig. 5c). This emergence of a strong population growth of large eukaryotic osmotrophs suggests that they may compete with heterotrophic bacteria for nutrients, thus shaping the carbon flux through the food web more than previously appreciated. Since they are larger than the average bacterium, eukaryotic osmotrophs can escape grazing by many micrograzers and directly transfer carbon to larger predators as zooplankton. Though we cannot elucidate the mechanism of potential competition between thraustochytrids and heterotrophic bacteria, possibilities include direct inhibition of bacterial growth by antimicrobial lipids^63^, niche separation in the degradation of different components of the organic matter, or efficient capture of organic matter by ectoplasmic nets directly from senescent *E. huxleyi* cells. More generally, our findings highlight that a complete understanding of the carbon flux in phytoplankton blooms requires deeper understanding of both the associated prokaryotic and eukaryotic microbial communities and the interactions between them^64^. More work is needed to fully establish the role of eukaryotic osmotrophs in the microbial loop, especially in the context of viral infections and the associated metabolome^65^.

Third, by relating ecosystem changes and biogeochemical processes to the varying degrees of active viral infection and lysis, we have shown that even moderate viral infection can significantly increase the per-cell production and release of extracellular carbon (Fig. 5d,e), both organic (TEP) and inorganic (PIC)^10,45,66^. The increase in TEP and PIC production per cell, either through a population level response or a specific metabolic remodeling of infected cells, could lead to elevated vertical carbon transport through aggregation and increase of the particle ballast. Enhanced production of TEP by infected cells not only increases the formation of sinking aggregates, but may also protect non-infected cells by trapping newly produced virions in sticky particles or can mask receptors needed for viral entry^12^. Alternatively, TEP could be involved in elevated virion production^67^ or the transport of virions to neighboring cells, in analogy to the human T-cells leukemia virus which encases itself in a host-derived carbohydrate-rich adhesive extracellular cocoon that enables its efficient and protected transfer between cells^68,69^. The increased production of PIC per cell is surprising since viral infection is thought to promote decalcification^70^. Nevertheless, higher turnover of coccolith through shedding and recalcification, or the production of thicker coccoliths, could be potential defense mechanisms, enabling lower viral adsorption and efficient removal of attached viral particles.

Our mesocosm experiment raises a fundamental question: how can quasi-identical starting points lead to such different bloom dynamics? There was no statistical difference in the early dynamics between the most and least infected communities in our experiments that would suggest a cause for increased or reduced susceptibility to viral infection. We therefore hypothesize that small-scale stochastic effects drive the tipping point of the evolving bloom ecosystem into different states. For instance, different replicates may have started with seed population containing slightly different fractions of susceptible *E. huxleyi* cells or different strains of its virus. In addition, the life cycle of EhV, and of giant viruses in general, remains largely unexplored; a deeper understanding of the diverse strategies that viruses can undertake to replicate and egress from the cells (through chronic release or cell bursting) is needed for a better comprehensive understanding of bloom dynamics. The observed population dynamics illustrate the inherent biological complexity of a natural bloom. Taken together, our results provide a strong evidence that viral infection does not only play an important ecological role as a principal cause of phytoplankton mortality, but also impact the fate of algal biomass, both by diverting carbon from bacteria toward larger eukaryotes and by potentially enhancing vertical export (Fig. 5). This refined assessment of viral impacts on the fate of carbon in the ocean helps bridge the scales between dynamic processes at the single cell, population, and biogeochemical levels.

## Methods

### Methods for data analysis in figures

All analyses in figures were performed using Mathematica 12.3 (Wolfram Research, Inc., Champaign, IL, USA).

#### Analysis in Fig. 1

C&D. To calculate integrated abundances of *E. huxleyi* cells and EhV, we first selected days for which all the bags had a non-null value. Values were then summed up to obtain the integrated abundance.

E&J. We computed a standard linear fit between the *E. huxleyi* total abundances and total EhV abundances for covered and uncovered bags separately. We followed the same procedure for the correlations in panel J and provide a comparison between different models in Supplementary Fig. 5.

#### Analysis in Fig. 2

A. The ASVs that were selected appeared at a relative abundance of at least 2% in at least 4 samples for the 0.2–2 µm 16S sequences and at least in 8 samples for the 2–20 µm 18S sequences. Abundances were concatenated for each time point and normalized by row, to have maximum relative abundance of 1 across all samples. ASVs were sorted by the position of their individual center of mass ${t}_{{CM}}$ defined by

1 $${t}_{{CM}}=\,\frac{\mathop{\sum}\limits_{i}{t}_{i}f({t}_{i})}{\mathop{\sum}\limits_{i}f({t}_{i})}$$

with *i* representing the different time points and *f(*${t}_{i}$*)* the relative abundance of the ASV. The same figure for the individual bags in shown in Supplementary Fig. 14 and Supplementary Fig. 15.

B. We selected 18S ASVs with a maximum relative abundance of at least 2% and observed in at least five samples. We averaged relative abundance across bags and then smoothed the time series with a moving average filter (width 2). Then, we grouped all ASVs into clusters based on their cosine distance using Mathematica’s FindClusters function and the KMeans method. The number of possible clusters ranged from 2 to 12, and the final number of clusters was decided using the silhouette method^71^. Only silhouette scores for 2 and 6 clusters were positive (between-cluster distance minus within-cluster distance).

D. We subset reads that map to either Flavobacteriales or Fhodobacterales, then renormalized within each class, taking the mean over bags. Results per bag are shown in Supplementary Fig. 9.

F. The turnover time was defined by the exponential rate *k* at which the Bray-Curtis similarity ${BC}(t)$ declined over time. To this end, for a given bag, we computed the Bray–Curtis similarity between the composition vector at a starting day *t’* with all following days *t*, giving a curve that declined roughly exponentially. For earlier starting days (for which the similarity curves declined the furthest), we found that the Bray–Curtis similarity never reached 0 but instead leveled out around ${{BC}}_{\infty }=0.05$ (due to ASVs that are constantly present in all the samples and maintain a minimal level of similarity between bags). Thus, we imposed an offset at$\,{{BC}}_{\infty }$ for all fits (using Mathematica’s FindFit function) with the function:

2 $${BC}(t)=(1-{{BC}}_{{{\infty }}}) \times {e}^{-k\left({t}^{{\prime} }-t\right)}+{{BC}}_{{{\infty }}}$$

The turnover is averaged over bags, showing the standard deviation as error bars in the figure.

G. To find differentially abundant ASVs, we first selected a subset of ASVs that had a maximum abundance of at least 10%, and performed Mann–Whitney U-Tests between the relative abundance values of a given ASV in the focal bag and all the other bags over all timepoints of the bloom’s demise. Correcting for multiple testing, we found four 16S ASVs that were differentially abundant in any of the bags, three of which were specific to bag 7, shown in Fig. 2g; and five 18S ASVs, two specific to bags 5 and 6 (*Rhizosolenia delicatula* and *Aplanochytrium*), one specific to bag 4 (*Pterosperma*), and two specific to bag 7 (*MAST-1C* and *Woloszynskia halophila*, shown in Fig. 2g).

H. The divergence between bags was calculated as follows: we first measured, for each bag, the Bray–Curtis distance between this given bag and all the other bags at the end of the experiment (Supplementary Fig. 13). In order to control for the existing differences between bags at the beginning of the bloom, Bray–Curtis distances were normalized according to the differences between bags at the starting day of the *E. huxleyi* bloom. As the exact starting days of the bloom is not clear, we normalized for starting days 11, 12, or 13. The plot shows averages with the standard deviation as error bars. For the 18S microbiome, we first removed reads that map to *E. huxleyi* to reduce bias toward bag 7 (which had by far the lowest *E. huxleyi* abundance, Fig. 1c).

#### Analysis in Fig. 3

A. Functional annotation of dominant 18S ASVs was based on manual literature search for the 100 most abundant 18S ASVs. Automatic annotation using the functional database created by^72^ gave qualitatively identical results but contained fewer organisms (covering about 50% of reads). The relative abundance of each trait was obtained by summing up the relative abundance of all the species harboring a specific trait. We used the annotations from^72^ to further subdivide heterotrophs into osmotrophs, saprotrophs, and other types of heterotrophy (e.g., grazing), ignoring ASVs with missing annotations.

D. Growth rates were computed by fitting a linear model to the log-transformed absolute abundances. For thraustochytrids, we measured growth rates until the abundances reached their maximum, i.e., for days indicated by solid lines in Fig. 3b. For bacteria in the 0.2–2 micron fraction, we measured growth rates during the bloom and demise of *E. huxleyi*, i.e., for the time period after day 15 until the final day, except for bag 4 (until day 22) and bag 7 (until day 18) to account for their different bloom and demise dynamics. For bacteria in the 2–20 micron fraction, we measured growth rates similarly, starting after day 10 until the final day, except bags 4 and 7 (until day 22).

E. To quantify the rate of change *k* of the biomass ratio of thraustochytrids to bacteria we fit a linear function to the log of biomass ratio from day 10 to the time point *t* where the ratio was maximal; for bag 7, this was day 18, for all others, day 23. We thus have:

3 $$\,{{\log }}\,{BR}\,(t)={kt}\,+\,{{\log }}\,{BR}\,(0)$$

#### Analysis in Fig. 4

C&D. Since TEP accumulates over time, it cannot be expressed as a weighted sum of phytoplankton abundances. Instead, we formulate the model as a recursive relation where TEP can be produced by *E. huxleyi*, naked nanophytoplankton, and picophytoplankton, and degraded or lost through sinking:

4 $${TEP}\left(t\right)=\left(1-d\right){TEP}\left(t-1\right)+{a}_{E}E\left(t\right)+{a}_{N}N\left(t\right)+{a}_{P}P\left(t\right),$$

The amount of TEP at time *t* is given by the fraction (1-*d*) of TEP at time *t*-1, where *d* corresponds to the fraction of TEP that is degraded between time points, plus the amount of TEP produced by the phytoplankton cells present at time *t* (or time *t*-1, which gives equivalent results). E, N, and P correspond to *E. huxleyi*, naked nanophytoplankton, and picophytoplankton, respectively. The parameter ${a}_{E}$ corresponds to the amount of TEP produced per *E. huxleyi* cell, reported in panel D. ${a}_{E}$ is set to be fixed through time, and different for each bag. This recursion can be solved to give an explicit expression for TEP(t):

5 $${TEP}\left(t\right)=\mathop{\sum }\limits_{{t}^{{\prime} }=0}^{t}{\left(1-d\right)}^{t-{t}^{{\prime} }}[{a}_{E}E\left({t}^{{\prime} }\right)+{a}_{N}N\left({t}^{{\prime} }\right)+{a}_{P}P\left({t}^{{\prime} }\right)].$$

This functional form was then used to perform a linear model fitting with the constraint ${a}_{i}\ge 0$ for various values of the parameter *d*. The best fit, defined by maximum ${R}^{2}$ over the resulting linear model, was used to fix *d* = 0.12. Our model considers that the fraction of non-calcified *E. huxleyi* cells in the nanophytoplankton counts is small.

Larger phytoplankton cells (>40 μm) filtered out from flow-cytometry measurements can also be a major source of TEP, despite low cell density. In order to verify this, FlowCam data was analyzed. None of the identified classes of larger phytoplankton (such as *Phaeocystis* or *Dinobryon*) increased in a systematic manner toward later stages of the bloom, explaining why larger phytoplankton were not included in the TEP model (Supplementary Fig. 24 and Supplementary Fig. 25).

E. Using the smFISH method that reports the proportion of infected *E. huxleyi* cells, we estimated the amount of TEP produced from infected cells. We first used the least infected uncovered bags (bags 1 and 3) as a baseline to fix model parameters such as how much TEP does a non-infected cell produce. We then split the *E. huxleyi* abundance into an uninfected subpopulation producing *T* TEP/cell as in the uninfected bags, and an infected subpopulation producing *I×T* TEP/cells. To define *I*, we combined the fixed model parameters (i.e., amount of TEP produced per cell from Fig. 4d for bags 1 and 3) with the measured fraction of infected cells. We adjusted the factor *I* = *4* to minimize deviation of the measure total TEP concentration from the model prediction including the two subpopulations. The same procedure was used for panel H, using the corresponding model for PIC.

F&G. To model the amount of PIC produced per cell we assume that the measured PIC only increases via new *E. huxleyi* coccoliths. The equivalent model for PIC reads

6 $${PIC}\left(t\right)=\left(1-d\right){PIC}\left(t-1\right)+{a}_{E}{{\max }}\left(E\left(t\right)-E\left(t-1\right)\right).$$

Where ${a}_{E}$ is the amount of PIC produced per cell, and displayed in panel G. Using the same procedure as for TEP, we obtain the best fit for *d* = 0.0075. Our PIC model assumes that all PIC production comes from *E. huxleyi*, supported by large occurrence of *E. huxleyi* cells observed in scanning electron microscopy (Supplementary Fig. 1).

### Methods for data collection

#### Mesocosm core setup

The mesocosm experiment AQUACOSM VIMS-Ehux was carried out for 24 days between 24th May (day 0) and 16th June (day 23) 2018 in Raunefjorden at the University of Bergen’s Marine Biological Station Espegrend, Norway (60°16′11 N; 5°13′07E). The experiment consisted of seven enclosure bags made of transparent polyethylene (11 m^3^, 4 m deep and 2 m wide, permeable to 90% photosynthetically active radiation) mounted on floating frames and moored to a raft in the middle of the fjord. The bags were filled with surrounding fjord water (day −1; pumped from 5 m depth) and continuously mixed by aeration (from day 0 onwards). Each bag was supplemented with nutrients at a nitrogen to phosphorus ratio of 16:1 according to the optimal Redfield Ratio (1.6 µM NaNO_3_ and 0.1 µM KH_2_PO_4_ final concentration) on days 0–5 and 14–17, whereas on days 6, 7 and 13 only nitrogen was added to limit the growth of pico-eukaryotes and favor the growth of *E. huxleyi* that is more resistant to phosphate limited conditions. Silica was not added as a nutrient source in order to suppress the growth of diatoms and to enhance *E. huxleyi* proliferation. Bags 5, 6, 7 were covered to collect aerosols and guarantee minimal contamination while sampling for core variables. Bags 1, 2, 3, 4 were sampled for additional assays such as metabolomics, polysaccharides profiling, and vesicles, which increase sampling time and potential for contamination.

#### Measurement of dissolved inorganic nutrients

Unfiltered seawater aliquots (10 mL) were collected from each bag and the surrounding fjord water in 12 mL polypropylene tubes and stored frozen at −20 °C. Dissolved inorganic nutrients were measured with standard segmented flow analysis with colorimetric detection^73^, using a Bran & Luebe autoanalyser. Data are available in ref. ^74^ and values for individual bags are plotted in Supplementary Fig. 26.

#### Measurement of water temperature and salinity

Water temperature and salinity were measured in each bag and the surrounding fjord water using a SD204 CTD/STD (SAIV A/S, Laksevag, Norway). Data points were averaged for 1–3 m depth (descending only). When this depth was not available, the available data points were taken. Data are missing for the fjord in days 0–1. Outliers were removed for the following samples: bag 1 at days 0, 4, 15; bag 7 at day 15. Data are available in ref. ^74^.

#### Flow cytometry measurements

Samples for flow cytometric counts were collected twice a day, in the morning (7:00 a.m.) and evening (8:00–9:00 p.m.) from each bag and the surrounding fjord, which served as an environmental reference. Water samples were collected in 50 mL centrifugal tubes from 1 m depth, pre-filtered using 40 µm cell strainers, and immediately analyzed with an Eclipse iCyt (Sony Biotechology, Champaign, IL, USA) flow cytometer. A total volume of 300 µL with a flow rate of 150 µL/min was analyzed with the machine’s software ec800 v1.3.7. A threshold was applied based on the forward scatter signal to reduce the background noise.

Phytoplankton populations were identified by plotting the autofluorescence of chlorophyll versus phycoerythrin and side scatter: calcified *E. huxleyi* (high side scatter and high chlorophyll), *Synechococcus* (high phycoerythrin and low chlorophyll), nano- and picophytoplankton (high and low chlorophyll, respectively). Chlorophyll fluorescence was detected by FL4 (excitation (ex): 488 nm and emission (em): 663–737 nm). Phycoerythrin was detected by FL3 (ex: 488 nm and em: 570–620 nm). Raw.fcs files were extracted and analyzed in R using ‘flowCore’ and ‘ggcyto’ packages and all data are available on Dryad^74^. In particular, the gating strategy was adapted to each day and each bag and individual plots for each days and each bag can be found in the Dryad link.

For bacteria and viral counts, 200 µL of sample were fixed with 4 µL of 20% glutaraldehyde (final concentration of 0.5%) for 1 h at 4 °C and flash frozen. They were thawed and stained with SYBR gold (Invitrogen) that was diluted 1:10,000 in Tris-EDTA buffer, incubated for 20 min at 80 °C and cooled to room temperature. Bacteria and viruses were counted and analyzed using a Cytoflex and identified based on the Violet SSC-A versus FITC-A by comparing to reference samples containing fixed bacteria and viruses from lab cultures. A total volume of 60 µL with a flow rate of 10 µL/min was analyzed. A threshold was applied based on the forward scatter signal to reduce the background noise. For plotting bacteria (Fig. 1h), a moving average of three successive days was used.

#### Enumeration of extracellular EhV abundance by qPCR

DNA extracts from filters from the core sampling (see above) were diluted 100 times, and 1 µL was then used for qPCR analysis. EhV abundance was determined by qPCR for the major capsid protein (*mcp*) gene: 5′-acgcaccctcaatgtatggaagg-3′ (mcp1F) and 5′-rtscrgccaactcagcagtcgt -3′ (mcp94Rv). All reactions were carried out in technical triplicates using water as a negative control. For all reactions, Platinum SYBER Green qPCR SuperMix-UDG with ROX (Invitrogen, Carlsbad, CA, USA) was used as described by the manufacturer. Reactions were performed on a QuantStudio 5 Real-Time PCR System equipped with the QuantStudio Design and Analysis Software version 1.5.1 (Applied Biosystems, Foster City, CA, USA) as follows: 50 °C for 2 min, 95 °C for 5 min, 40 cycles of 95 °C for 15 s, and 60 °C for 30 s. Results were calibrated against serial dilutions of EhV201 DNA at known concentrations, enabling exact enumeration of viruses. Samples showing multiple peaks in melting curve analysis or peaks that were not corresponding to the standard curves were omitted. Data are available in ref. ^74^. A comparison of viral counts based on flow-cytometry and qPCR is shown in Supplementary Fig. 2.

#### FlowCam analysis

Samples for automated flow imaging microcopy were collected once a day in the morning (7:00 a.m.) from each bag and the surrounding fjord, which served as an environmental reference. Water samples were collected in 50 mL centrifugal tubes from 1 m depth, kept at 12 °C in darkness, and analyzed within 2 h of sampling, using a FlowCAM II (Fluid Imaging Technologies Inc., Scarborough, ME, USA) fitted with a 300 µm path length flow cell and a 4× microscope objective. Images were collected using auto-image mode at a rate of 7 frames/second. A sample volume of 10 mL was processed at a flow rate of 0.7 mL/min. Individual objects within each sample were clustered and annotated using the Ecotaxa platform^75^. Absolute counts for major groups, including the most abundant ciliate category Ciliophora U04, were then exported and normalized by the individual amount of water volume processed for each sample.

Data are available under “Flowcam Composite Aquacosm_2018_VIMS-Ehux” project on Ecotaxa.

#### Scanning electron microscopy

50 ml of water samples from bags or fjord were collected on polycarbonate filters (0.2 µm pore size, 47 mm diameter, Millipore). The filters were air dried and stored on petri-slides (Millipore) at room temperature. Prior to observation, a small fraction of the filter was cut and coated with 2 nm of iridium using a Safematic CCU-010 coater (Safematic GMBH, Switzerland). Samples were observed on a Zeiss Ultra SEM that was set at a working distance of 6.2 ± 0.1 mm, an acceleration voltage of 3.0 kV and an aperture size of 30 mm. The secondary electron detector was used for image acquisition.

#### Paired dilution experiment

Phytoplankton growth and microzooplankton grazing rates were estimated using the dilution method^76,77^. A slightly modified version of the method was used with only one low dilution level (20%) and an undiluted treatment used^78^. Rates calculated using this method are considered conservative but accurate when compared with those using multiple dilution levels and a linear regression. Water from bags 1–4 was collected using a peristaltic pump at ~1 m depth and mixed into a 20 L clean carboy. Water was screened through a 200 µm mesh to remove larger mesozooplankton. The collected water was shaded with black plastic and returned to shore. Dilution experiments were set-up in a temperature-controlled room, set to ambient water temperature (±2 °C). Particle-free diluent (FSW) was prepared by gravity filtering whole seawater (WSW) through a 0.45 µm inline filter (PALL Acropak™ Membrane capsule) into a clean carboy. To the FSW, WSW was gently siphoned at a proportion of 20%. The 20% dilution and 100% WSW treatments were prepared in single carboys and then siphoned into triplicate 1.2 L Nalgene™ incubation bottles. To control for nutrient limitation, additional triplicate bottles of 100% WSW were incubated without added nutrients (10 µM nitrate and 1 µM phosphate). The incubation bottles were incubated for 24 h in an outdoor tank maintained at in-situ water temperatures by a flow-through system of ambient seawater. Bottles could float freely, and the seawater inflow caused gentle agitation throughout the 24 h period. A screen was used to mimic light conditions experienced within the mesocosm bags.

To quantify viral mortality, we used the paired dilution method^79^ which involves setting up an extra low dilution level (20%) containing water filtered through a tangential flow filter (TFF) of 100 kDå to remove viral particles. During this experiment, TFF water was produced 1–2 days prior to the dilution experiment, to ensure the chemical composition of the water was as similar as possible, and experiments could be set up in a timely manner.

At T0 hours and T24 hours from all dilution experiments, sub-samples were taken for the determination of chlorophyll-*a* and flow cytometry. For chlorophyll-*a*, 100–150 mL of seawater was filtered under low vacuum pressure through a 47 mm Whatman GF/F filters (effective pore size 0.7 µm), and then extracted in 7 mL of 97% methanol at 4 °C in the dark for 12 h. All chlorophyll readings were conducted on a Turner TD700 fluorometer^80^. Methanol blanks were included, and all samples were corrected for phaeophytin using a drop of 10% hydrochloric acid and then reading the sample again^81^.

Water samples (2 × 1 mL) for flow cytometry were taken at T0 and T24 of dilution experiments for the determination of phytoplankton abundances. Water samples were taken in triplicate from T0, and from each bottle at T24. Samples were immediately fixed in 20 µL of glutaraldehyde (final concentration <1%), gently inverted and then stored at 4 °C for up to 2 h. Samples were then flash frozen in liquid nitrogen and kept at −80 °C until analysis. Samples were thawed and run at a high flow rate (104–108 µL min^−1^) on a FACSCalibur (Becton Dickinson, East Rutherford, USA) for 1–5 min, based on the number of events triggered per second. Phytoplankton groups were differentiated into four groups; picoeukaryotes, nanoeukaryotes, *Synechococcus*, and *E. huxleyi* as explained above.

The apparent growth rates (*k*) of the total phytoplankton community (chlorophyll-*a*) and individual phytoplankton groups was calculated using the equation:

7 $$k=1/t\,{{{{{\rm{ln}}}}}}({C}_{t}-{C}_{0})$$

Where t = incubation time in days, *C*_*t*_ and *C*_*0*_ are the final and initial concentrations of chlorophyll-*a* or cell counts respectively.

Grazing and growth rates were calculated as in Eqs. 4 and 5 of Morison and Menden-Deuer (2017). Grazing (*g*) was calculated as:

8 $$g=({k}_{d}-{k}_{1})/(1-x)$$

Where, *k*_*d*_ is the average growth rate in the diluted treatment (20%) and *x* is the fraction of WSW, and *k*_*1*_ is the average growth rate in 100% WSW with nutrient addition. Once grazing rates were calculated, the intrinsic growth rate (*µ*) is calculated using *k*_*1*_, which is the average growth rate without nutrients added:

9 $${\mu }=g+{k}_{1}$$

Paired *t*-tests were conducted to determine significant differences (*p* < 0.1) between 100% WSW with and without nutrient additions. If no difference was found, the growth rates were pooled for calculations, otherwise calculated as above. Significant grazing rates were also determined through paired *t*-tests (*p* < 0.1) between 100% WSW and diluted treatments (20% WSW). Viral lysis was calculated as above for grazing, and if detected we also checked for a significant difference (*p* < 0.1) between diluted treatments with FSW and TFF waters to determine if the technique was sensitive enough to determine differences. On dates when viral lysis was determined, the intrinsic growth rate was calculated using both grazing and viral lysis rates. Results are shown in Supplementary Fig. 6.

#### Core microbiome harvesting, sequencing, and annotation

Every day, between 1 and 2 L of water samples of each bag and fjord water were pre-filtered at 200 µm, then filtered sequentially through 20 µm and 2 µm, and finally 1–2 L filtrate was filtered through 0.22 µm hydrophilic polycarbonate filters (Isopore, 47 mm; Merck Millipore, Cork, Ireland). Filters were immediately flash frozen in liquid nitrogen and stored at −80 °C until further processing. DNA was extracted from the 2 to 20 µm filters using the DNeasy PowerWater kit (Qiagen, Hilden, Germany) according to the manufacturer’s instructions. 0.2 µm filters were extracted using DNAdvance Kit (Beckman-Coulter, Brea, USA).

The bacterial community was sequenced using the EMP 16S amplicon protocol and 515F-806R primers^82^ at the Environmental Sample Preparation and Sequencing Facility (ESPSF), which is located in the Argonne National Laboratory. Degeneracy was added to the 515 F primer to reduce bias against Crenarchaeota/Thaumarchaeota (also called 515F-Y^83^) and to the 806 R primer to minimize the bias against the SAR11 clade (806R^84^). The primer sequences without the linker, pad, barcode, or adapter are as follows: 5′ -GTGYCAGCMGCCGCGGTAA - 3′(515F-Y) 5′—GGACTACNVGGGTWTCTAAT - 3′ (806 R). ASVs were called using DADA2^85^ with standard parameters. Taxonomic identity mapping was performed using RDP classifier^86^. The average sequencing depth per sample was about 27000 ± 6200 (min 5500, max 49500 reads). Reads were normalized to the total amount of reads within each sample to convert them into relative abundance. Prior to further analysis, all reads that map to chloroplasts were removed (corresponding to up to 5% of all reads during the first bloom and 15% of all reads during the *E. huxleyi* bloom). For the particle-attached community, the analysis focused on bags 2, 3, 4, and 7.

For the 18S sequencing of the DNA extracts from the 0.2 µm to 2 µm filters, the V4 region of the 18S rDNA sequence was amplified using the TAREuk454FWD1 (5′‐CCAGCA(G/C)C(C/T)GCGGTAATTCC‐3′) from^87^ and a modified V4Rev_Piredda (5′—ACTTTCGTTCTTGATYRATGA - 3′) from^88^ in order to identify *E. huxleyi*, combined with CS1 and CS2 Illumina adaptors. We used the following PCR mix: 12.5 µL of Buffer myTAQ HS 2X Mix, 1 µL of each primer 0.4 µM final concentration, 0.75 µL of DMSO 3%, 8.75 µL of ultrapure water, 1 µL of DNA template. We used the following PCR conditions: initial denaturation of 2 min at 95 °C followed by 10 cycles of 10 s 95 °C, 30 s 53 °C, 30 s 72 °C then 15 cycles of 10 s 95 °C, 30 s 48 °C, 30 s 72 °C, final elongation of 10 min at 72 °C. PCR products were prepared for Illumina sequencing on a MiSeq 2 × 250. Fastq files were then cleaned and amplicon sequencing variants determined using the DADA2 pipeline^85^, annotated with the PR2 database^89^ and analyzed using the “phyloseq” package in R^90^.

Data has been deposited under NCBI Bioproject PRJNA694552: 16S data is available under Biosample SAMN17576248 and 18S data is available under Biosample SAMN20295136.

#### ddPCR quantification

*Thraustochytrids*: Digital droplet PCR (Bio-Rad, Hercules, USA) was performed on 2 µm mesocosm filters of days 2, 8, 14, 16, 18, 20, 23 of each bag including the fjord, to assess the absolute concentration of thraustochytrids. For VICE-cruise samples^45^, representative samples of each bloom phase were chosen (Casts 23, 27 for Post Infection; Casts 77, 79 for Late Infection, Casts 52, 63, 68, 72 for EI and Casts 84, 92, 97 for EIr).

Primers targeting the 18S rDNA gene of thraustochytriaceae were used^91^ with forward primer SYBR-ThF 5′-GGATCGAAGATGATTAGATACCA-3′ and reverse primer SYBR-ThR 5′- GACTTTGATTTCTCATGTGC -3′. Primers were checked for specificity in PR2^89^. Sample mix consisted of 10 µL of 2X QX200 ddPCR EvaGreen supermix, 1 µL of 2uM forward primer, 1 µL of 2 µM reverse primer, 5 µL of water and 5 µL of the DNA sample. To load the optimal amount of DNA, DNA extractions were diluted 1:10 and DNA concentration was measured using a Qubit dsDNA HS Assay Kit (Invitrogen, Waltham, USA). Depending on the concentration, between 1 and 5 µL of extracts were completed to a total of 5 µL with ultra-pure water, and used in the final ddPCR reaction. Less than 80 ng of DNA was used for each reaction. From the final mix of 22 µL, 20 µL of each sample were loaded in the DG8 Cartridge and inserted in the QX200 droplet generator. Each cartridge contained a negative control containing the ddPCR mix with 5 µL of water. After droplet generation, samples were transferred to a 96 well-plate and inserted in a C1000 Touch thermal cycler. The following cycle was used: 95 °C 5 min, followed by 40 cycles of 96 °C for 30 s, 58 °C for 1 min, 4 °C 5 min, 90 °C 5 min and infinite hold at 4 °C. After thermal cycling, the 96-well plate was read in the QX200 Droplet Reader and results analyzed using the Quantasoft software.

Quantasoft provides a final concentration of target copies/µL of ddPCR reaction. For mesocosm samples, we first calculated the total amount of target copies in 20 µL of ddPCR reaction and normalized it by the amount of sea water that was sampled, to obtain a final concentration of target copies/mL of sampled sea water. To convert 18S copies/mL into cell/ml, we estimated the amount of 18S copies per thraustochytrid cell. The number of 18S rDNA copies/cell was calculated based on the relationship between genome size and copy number recently published in ref. ^92^. Published thraustochytrid genomes range between 38.7 Mb^93^ and 43 Mb^94^. Using the regression equation on log transformed data with an average thraustochytrid genome size of 40 Mb, we obtain f(x) = 0.6607(log(40)) + 0.7508 = 1.809 with f(x) the log value of total 18S copies. We therefore obtain that the estimated 18S copy number in thraustochytrids cells is 10^1.809^ = 64 copies. The thraustochytrid biomass was estimated based on a value of 1.65 × 10^−10^ g of C/cell^44^. The bacterioplankton biomass was estimated based on a value of 10 × 10^−15^ g C/cell^95^, using abundance counts from the flow cytometer. A detailed calculation for each sample is available in Supplementary Data 1, 2. For cruise samples, we report copies per ng of extracted DNA.

#### Sanger sequencing of thraustochytrids from environmental samples

To identify thraustochytrid species from the mesocosm, DNA extracts from June 16th 2018 (Day 23) of the 2–20 µm size fraction from bag 2, bag 4, bag 5, and bag 7 were used. To identify thraustochytrids from an open ocean bloom, DNA extracts from the NA-VICE Cruise Cast 79^45^, 28 m depth was chosen for its high concentration of thraustochytrids based on ddPCR.

DNA from each sample was used as a template in PCR reactions with the primer 18S-F^96^ and LABY-Y^97^ (~1400 bp product). PCR reactions were made with Platinum Taq DNA Polymerase reagents (Invitrogen, Waltham, USA) as follows: 5 µl 10× Platinium Taq buffer, 1 µl 10 mM dNTPs, 1 µl 10 µM of forward and reverse primers, 1.5 µl 50 mM MgCl_2_, 38.3 µl water; 0.2 µl Platinum Taq polymerase; and 2 µl template DNA. The PCR program was 35 cycles of 94 °C for 30 s, 50 °C for 1 min, and 72 °C for 2 min, followed by a final extension at 72 °C for 10 min as in ref. ^98^. Reaction products were examined by agarose gel electrophoresis. PCR products were directly cleaned with the Wizard SV Gel and PCR Clean-up System (Promega, Madison, USA) and analyzed by Sanger sequencing using four different primers: 18S-F^96^, LABY-A^97^, LABY-Y^97^ and LABY-ARev^98^. Chromatograms were cleaned and assembled using DNASTAR software, with the Sanger Analysis and Assembly program. Assembled sequences deposited on NCBI with accession numbers MZ562737, MZ562738, MZ562739, MZ562740, MZ562741.

For phylogeny, obtained sequences were blasted on NCBI. 50 similar sequences were obtained, and *Oblongichytrium* and *E. huxleyi* 18S sequences were chosen as outgroup. We generated an alignment in mafft, keeping only sequences longer than 1000 bp, leaving 32 sequences in the final alignment. A neighbor-joining tree was performed on conserved sites (866 bp) with Jukes-Cantor model and 1000 bootstraps. The tree was exported in Newick format, and edited in Illustrator.

#### Particle-attached bacteria quantification using 16S qPCR

To quantify bacteria by qPCR, we used primers targeting the V5–V6 of bacterial 16S rRNA and designed to exclude chloroplastic 16S (799 F: 5′-AACMGGATTAGATACCCKG-3′; 1192 R: 5′-ACGTCATCCCCACCTTCC-3′, from^99,100^. For 100 reactions we prepared the master mix containing 600 μL of Platinum SYBR Green qPCR SuperMix-UDG with ROX (Invitrogen), 4.5 μL of each 100uM primers stock (0.4 μM final concentration) and 291.25 μL of ultra pure water. Each reaction contained 7.5 μL of the master mix, and 2.5 μL of DNA template, for a final volume of 10 μL per reaction.

Bacteria were quantified on the 20 μm size fraction of bags 2, 3, 4, 7 for which we have 16S amplicon sequencing (1:100 dilution). For the 2 μm size fraction, we used the same filters that were used to quantify Labyrinthulomycetes in Fig. 3 (1:100 dilution). Triplicates were conducted for each DNA template. Triplicates of ultra pure water were used as negative control.

Calibration curve: given that our samples contain a large amount of diversity, we used microbiome communities directly coming from the mesocosm experiment. During the mesocosm experiment, a bacterial glycerol stock was made from bag 4 on day 21 (2018.06.14). A small amount of this glycerol stock was propagated on conditioned media of exponentially growing *E. huxleyi* and a new glycerol stock “GS12” was made. Conditioned media was obtained by filtering the culture on 0.45 μm Stericups to discard cells. For the qPCR calibration curve, a small amount of the GS12 glycerol stock was incubated overnight at 27 degrees in 4 mL of marine broth. On the next day, 750 μl of the bacterial culture was pelleted for 1 min at 13000 rpm and resuspended in 1.2 mL of filtered seawater. From this, we diluted 100 μL into 900 μL of filtered seawater to create the GS12 1:10 dilution and fixed 100 μL of this mix with 2 μL of glutaraldehyde to quantify bacteria with flow-cytometry. The remaining 900 μL were filtered on the 0.2 μm Swinnex, flash frozen and extracted using the DNeasy PowerWater Kit (Qiagen) with final elution in 200 μL, to conduct the same procedure as the mesocosm filters. Six 1:10 serial dilutions were performed, leading to a calibration curve of seven points. Calibration curves were run in triplicates.

qPCR was performed in 384 well plates on Quantstudio5 (Thermo Fisher Scientific) with the following condition: initial 20 s denaturation and enzyme activation at 95 °C followed by 40 cycles of denaturation at 95 °C and 20 s annealing and extension at 60 °C. Results were analyzed using the QuantStudio Design and Analysis Software v1.5.1. All calculations for qPCR quantification on 2 μm and 20 μm filters are detailed in Supplementary Data 3, 4.

#### Transparent exopolymer (TEP)

*TEP* concentration was determined following the spectrophotometric method^101^. Duplicate samples (50–200 mL) were filtered onto 25 mm diameter 0.4 µm pore size polycarbonate filters (DHI, San Francisco, USA) using a constant low filtration pressure (~150 mmHg). Immediately, the filters were stained with an Alcian Blue solution (500 µL, 0.02%, pH 2.5) for 5 s, and rinsed with MilliQ water. Duplicate blanks (empty filters) were stained with every batch of samples and all filters were stored frozen (−20 °C) in 2 mL Eppendorf tubes until further processing. Dye extraction of all filters was done by soaking in 5 mL of 80% sulfuric acid for 3 h, shaking them intermittently. Absorbance of samples and blanks was measured against MilliQ water at 787 nm using the Varian Cary 100 Bio, and the mean absorbance of daily blank filters was subtracted from each batch of samples. The staining solution was calibrated following the original method of^101^ with a xanthan gum standard and TEP concentration is reported in micrograms of xanthan gum equivalents per liter (µg XG eq/L).

#### Estimated pool of organic carbon derived from E. huxleyi

The estimated amount of organic carbon derived from *E. huxleyi* was calculated as follows. The volume *V* of an *E. huxleyi* cell was calculated based on a sphere of radius *R* = 2.5 µm using the formula

10 $$V=\frac{4}{3}\pi {R}^{3} \sim 65.4498{{{{{\rm{\mu m}}}}}}^3.$$

The carbon content for one *E. huxleyi* was calculated by using a volume to carbon conversion factor of 220 fg C/µm^3^ as in^102^ leading to an estimate of 14,398 fg C/cell or 14.398 pg C/cell. We then estimated a loss of 19,050 *E. huxleyi* cells/ml/day, which corresponds to the difference in average abundances between day 17 (57,000 cells/ml) and day 19 (18,900 cells/ml). This corresponds to a loss of 274,281 pg C/ml/day or 274.3 ng C/ml/day or 274 µC/L/day.

#### Particulate organic carbon (POC) and nitrogen (PON), and particulate inorganic carbon (PIC)

For POC analyses, seawater (150–1000 mL) was filtered through combusted (4 h, 450 °C) GF/F glass fiber filters (Whatman, Maidstone, UK) and filters were frozen at −20 °C until processed. Prior to analysis, the filters were thawed in an HCl-saturated atmosphere for 48 h to remove inorganic compounds and dried at 80 °C for 24 h^103^. Then the filters were dried and analyzed with an elemental analyzer (Perkin-Elmer 2400 CHN, Perkin-Elmer, Waltham, USA). For total particulate carbon (TPC) and PON the same procedure was followed except for the filter exposure to HCl-saturated atmosphere. PIC concentration was obtained subtracting POC from TPC values.

#### Coomassie stainable particles (CSP)

CSP concentration was determined by spectrophotometry following^104^. Duplicate samples (60–200 mL) were filtered onto 25 mm diameter 0.4 µm pore size polycarbonate filters (DHI) using a constant low filtration pressure (~150 mmHg). The samples were immediately stained with 1 mL of Coomassie Brilliant Blue (CBB-G 250) solution (0.04 %, pH 7.4) for 30 s, prepared daily with filtered 0.2 µm fjord water collected at the beginning of the experiment, and rinsed three times with MilliQ water. Duplicate blanks (empty filters) were stained with every batch of samples and all filters were stored frozen (−20 °C) in 2 mL Eppendorf tubes until further processing. Dye extraction of all filters was performed by soaking them in 4 mL of extraction solution (3% SDS in 50% isopropyl alcohol) for 2 h at 37 °C, shaking them every 30 min. Absorbance of samples and blanks was measured against MilliQ water at 615 nm (Varian Cary 100 Bio), and the mean absorbance of daily blank filters was subtracted from each batch of samples. The staining solution was calibrated with a bovine serum albumin standard and CSP concentrations are expressed accordingly in micrograms of bovine serum albumin equivalents per liter (µg BSA eq/L).

#### Chlorophyll a (Chl a)

Samples (100–250 mL) for fluorometric Chl *a* analysis were filtered on glass fiber filters (GF/F, 25 mm diameter, Whatman, Maidstone, UK) and stored at −20 °C until analysis. Pigments were extracted with 90% acetone at 4 °C in the dark for 24 h. Fluorescence of extracts was measured, and corrected for phaeopigments, with a calibrated Turner Designs fluorometer^105^.

#### Dissolved organic carbon (DOC)

For DOC determination, 30 mL samples of filtered sea water (GF/F, Whatman, Maidstone, UK) were collected in acid-cleaned polycarbonate bottles, and stored in the dark at −20 °C until analysis. They were analyzed with a TOC-LCSV (Shimadzu, Kyoto, Japan), with MilliQ water as a blank, potassium hydrogen phthalate as the calibration standard, and deep Sargasso Sea water as the reference (Hansell Laboratory. University of Miami, RSMAS). Each sample was injected repeatedly 4–5 times, until at least 3 reads yielded a relative standard deviation lower than 3%.

#### Polysaccharide analysis of particulate organic matter

A peristaltic pump and tubings with a 200 μm mesh were used to sample between 25 and 100 L of water from the enclosures, which was subsequently filtered through pre-combusted 0.7 μm GF/F filters (Whatman, Maistone, UK) to harvest particular organic matter (POM).

Polysaccharide extraction: For the POM samples, 7 circular filter sections (11.2 mm diameter) were punched out from each GF/F filter and transferred into a 2 ml tube. Polysaccharides were sequentially extracted with: MilliQ water, 50 mM EDTA pH 7.5 and 4 M NaOH with 0.1% w/v NaBH_4_. For each of the extracting solvents the following was performed: 400 µl of solvent were added to the tubes containing the filter pieces, vortexed them briefly and tubes were then incubated 2 h at 650 rpm (MilliQ at 60 °C and the other two solvents at room temperature). Samples were spun down at 6000 × g for 10 min at 15 °C. Extracts (supernatants) were collected in 1.5 ml tubes. The pellets and filter pieces were resuspended in the next extracting solvent using the same extraction procedure as depicted above.

Carbohydrate microarray analysis: All POM polysaccharide extracts were added into wells of 384-microwell plates. For each extract a twofold dilution followed by a fivefold dilution was performed in printing buffer (55.2% glycerol, 44% water, 0.8% Triton X-100). Plates containing the samples were spun down at 3500 × g for 10 min at 15 °C to get rid of bubbles. The content of the plates was printed onto nitrocellulose membrane with a pore size of 0.45 µm (Whatman, Maidstone, UK) using a microarray robot (Sprint, Arrayjet, Roslin, UK) under controlled conditions of 20 °C and 50% humidity. A printing replicate was included for each sample. Once printed, each single microarray was individually probed with one glycan-specific monoclonal antibody for microarray probing^55^. The developed arrays were scanned at 2400 dots per inch and binding of each probe (probe signal intensity) against each spotted sample was quantified using the software Array-Pro Analyzer 6.3 (Media Cybernetics, Rockville, USA). Briefly, array data analysis was performed as follows^55^: for each extract the mean antibody signal intensity was calculated. The highest mean signal intensity detected in the data set was set to 100 and all other values were normalized accordingly. Controls for the extraction solvents indicated no unspecific binding to any of the probes and controls for the anti-rat alkaline phosphatase-conjugated secondary antibody presented no unspecific binding to any of the samples and a cut-off of 5 arbitrary units was applied.

### Reporting summary

Further information on research design is available in the Nature Portfolio Reporting Summary linked to this article.

## Supplementary information

Supplementary Information

Description of Additional Supplementary Files

Supplementary Data 1-4

Reporting Summary

## Data Availability

All data needed to evaluate the conclusions in the paper are present in the paper and clearly indicated in the Methods. Flow cytometry, nutrient, and temperature data are available in Dryad: 10.5061/dryad.q573n5tfr. Flowcam data is available on Ecotaxa under the project “Flowcam Composite Aquacosm_2018_VIMS-Ehux” (https://ecotaxa.obs-vlfr.fr/prj/2501). Sequencing data has been deposited under NCBI Bioproject PRJNA694552: 16S data is available under Biosample SAMN17576248 and 18S data is available under Biosample SAMN20295136. The PR2 database can be found on Zenodo: zenodo.org/record/5031733. Assembled sequences deposited on NCBI with accession numbers MZ562737, MZ562738, MZ562739, MZ562740, MZ562741. All data used to produce figures are available in the Source Data file. Source data are provided with this paper.

## Source data

Source Data

## Code availability

The code for all the analyses is available on Mendeley at 10.17632/k33xvnxhdb.1 (https://data.mendeley.com/datasets/k33xvnxhdb/1).
