# Supplementary material for: Viral infection switches the balance between bacterial and eukaryotic recyclers of organic matter during coccolithophore blooms
Source: Nat Commun. 2023 Jan 31;14:510. doi: 10.1038/s41467-023-36049-3 (PMC9889395; doi:10.1038/s41467-023-36049-3)
Supplement: Supplementary file 1 — Supplementary Information [file 41467_2023_36049_MOESM1_ESM.pdf]

Supplementary Figures

Viral infection switches the balance between bacterial and eukaryotic recyclers of organic matter during coccolithophore blooms

Flora Vincent[§1#], Matti Gralka[§2$], Guy Schleyer[1], Daniella Schatz[1], Miguel Cabrera-Brufau[3], Constanze Kuhlisch[1], Andreas Sichert[2,4], Silvia Vidal-Melgosa[4,8], Kyle Mayers[5], Noa Barak-Gavish[1], J.Michel Flores[6], Marta Masdeu-Navarro[3], Jorun Karin Egge[7], Aud Larsen[5,7], Jan-Hendrik Heheman[4,8], Celia Marrasé[3], Rafel Simó[3], Otto X. Cordero[2], Assaf Vardi[1*]

Affiliations:

[1] Department of Plant and Environmental Sciences, Weizmann Institute of Science, Rehovot, 7610001, Israel

[2] Department of Civil and Environmental Engineering, Massachusetts Institute of Technology, Cambridge, 02145, MA

[3] Institut de Ciències del Mar, CSIC, Barcelona, 08003, Spain

[4] Max Planck Institute for Marine Microbiology, Bremen, 28359, Germany

[5] NORCE Norwegian Research Centre, Bergen, 5008, Norway

[6] Department of Earth and Planetary Science, Weizmann Institute of Science, Rehovot, 7610001, Israel.

[7] Department of Biological Sciences (BIO), University of Bergen, Bergen, 5020, Norway

[8] Center for Marine Environmental Sciences (MARUM), University of Bremen, Bremen, 28359, Germany

[§]These authors contributed equally to this work

[#]Present address: Developmental Biology Unit, European Molecular Biological Laboratory, Heidelberg, 69117, Germany

[$]Present address: Systems Biology Lab, Amsterdam Institute for Life and Environment (A-Life)/Amsterdam Institute of Molecular and Life Sciences (AIMMS), Vrije Universiteit Amsterdam, Amsterdam, 1081HV, The Netherlands

*Corresponding author: assaf.vardi@weizmann.ac.il

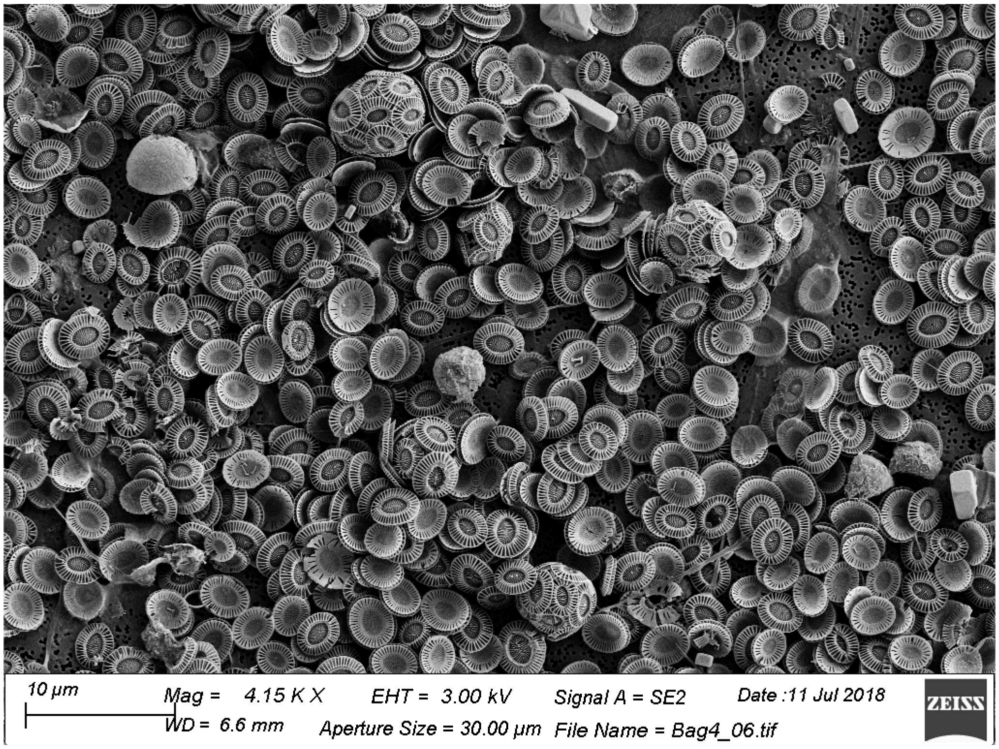

Supplementary Fig. 1: Scanning electron microscopy picture of Bag 4 on day 23.

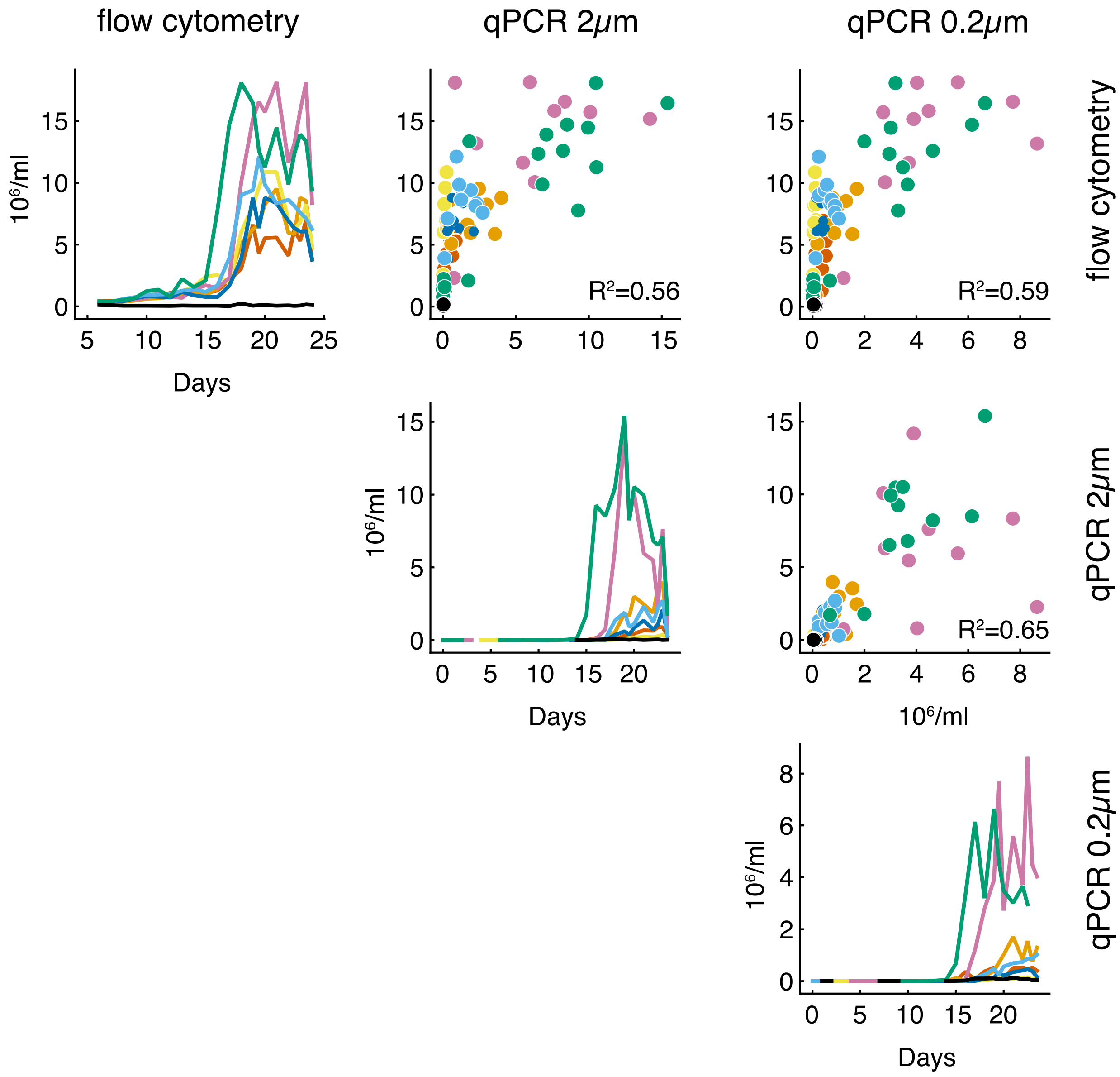

Supplementary Fig. 2: Cross comparison of viral quantification based on flow-cytometry versus qPCR of the *major capsid protein* on the 0.2-2 µm and 2-20 µm size fractions.

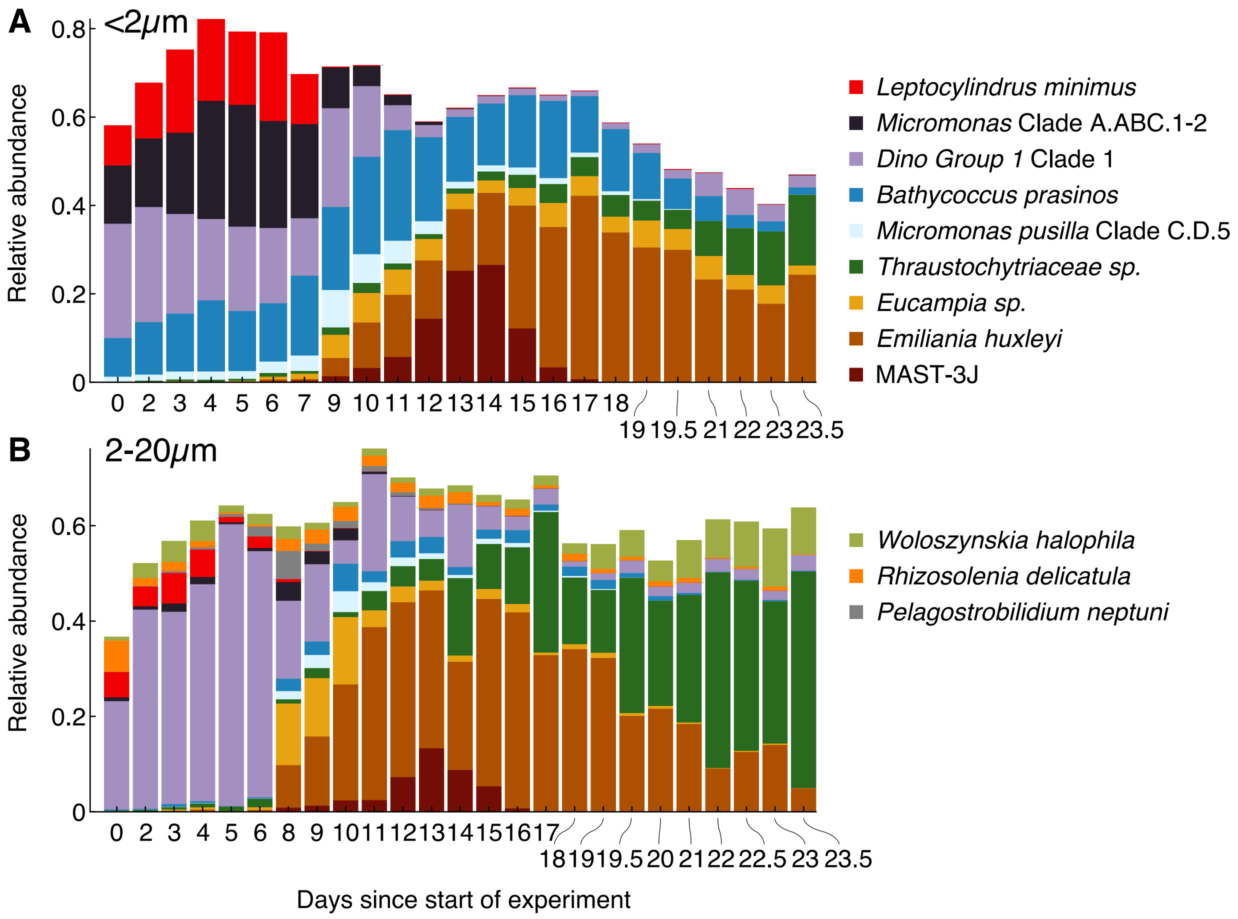

Supplementary Fig. 3: Stacked relative abundance of the twelve most abundant taxa in the 18S reads in the **(A)** 0.2-2μm and **(B)** 2-20μm size fractions. The large size fraction is entirely dominated by dinoflagellates for the first 6 days, whereas the 0.2-2μm fraction also features high abundances of small phytoplankters like *Leptocylindrus*, *Micromonas*, and *Bathycoccus*.

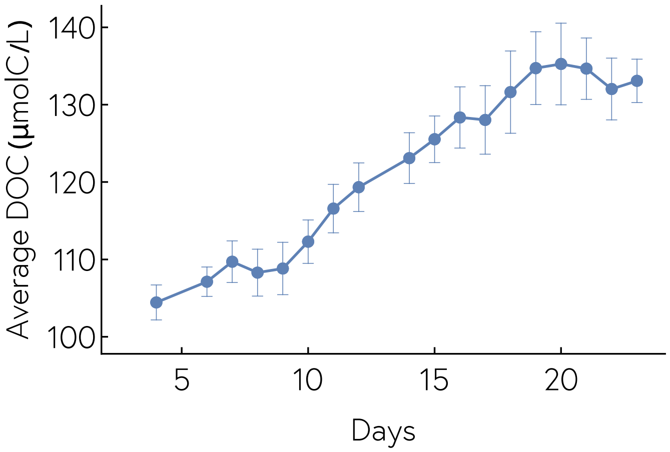

Supplementary Fig. 4: Dissolved organic carbon (DOC) concentration through time. Concentrations are averaged across all bags (n=7) and smoothed across three days with bars representing standard deviation.

Population	Model 1: pop ~ cover	Model 2: pop ~ EhV	Model 3: pop ~ EhV|cover
E. huxleyi	0.049711318	0.035555632	**0.005686443**
ciliates	0.704364851	0.023702313	**0.008848701**
pico-phytoplankton	0.068557395	0.159123307	0.167169489
naked nano-phytoplankton	0.063871099	0.619155409	0.143664902
bacteria	0.90886486	0.155865901	0.143110247

Supplementary Fig. 5: Table of correlation p-values that predict cell abundances for major groups according to three different linear models. The predictors for cell abundances are either the presence/absence of a cover (Model 1), the abundance of EhV (model 2), a combination of both (model 3, that first corrects for cover and then incorporates EhV abundance). *E. huxleyi* and ciliates abundances can be confidently predicted by using Model 3. Bold numbers represent significant correlations.

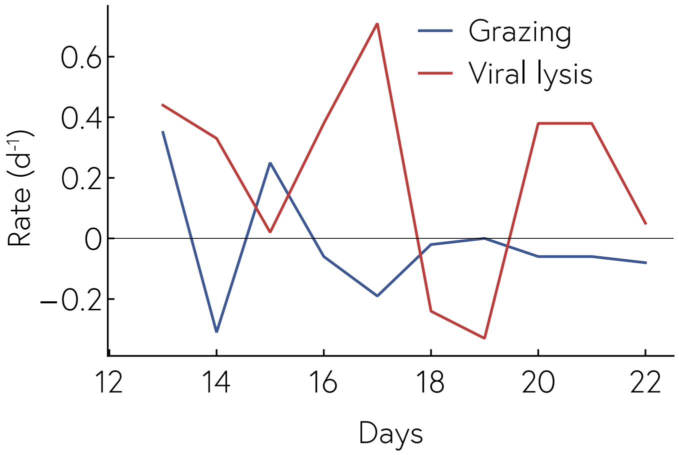

Supplementary Fig. 6: Rates of grazing (blue) and viral lysis (red) measured by paired dilution assay through time, using a mixture of water from bags 1-4. Briefly, the grazing assay measures rates of grazing and viral infection by evaluating phytoplankton growth rate between 0- and 24-hour incubation in bottles filled with either whole sea water (phytoplankton growth is limited by grazing or viral infection), either whole sea water diluted with 0.45µm filtered seawater (diluted effect of grazing but not of viruses as filtered seawater still contains viruses), or whole seawater diluted with TFF viral-free water (diluted effect of grazing and viruses). See Methods for calculation of rates.

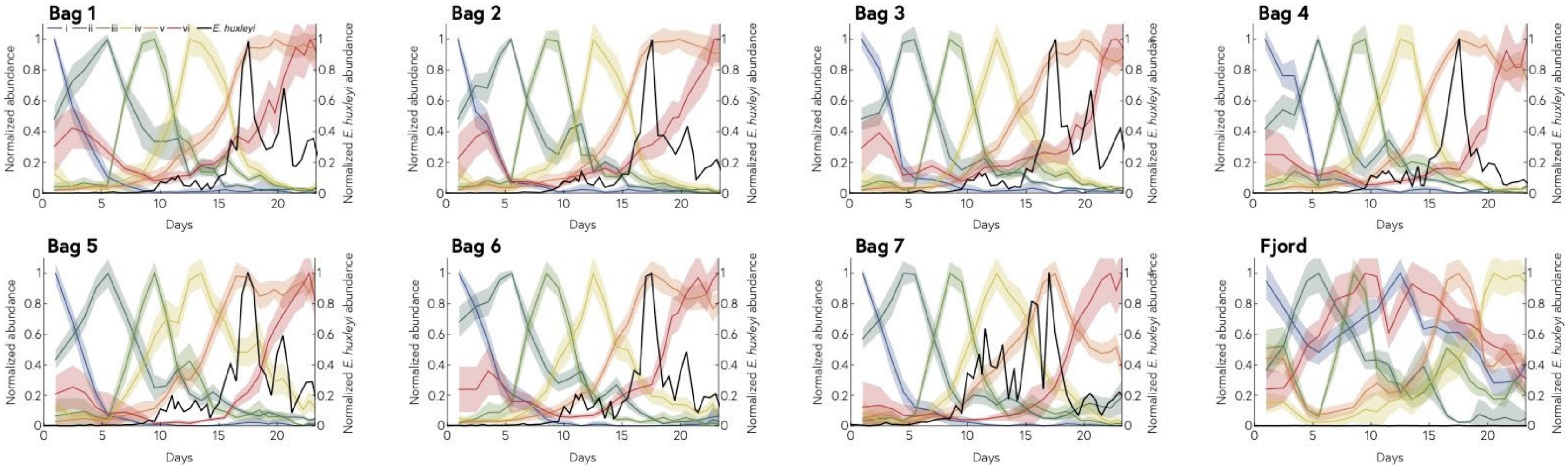

Supplementary Fig. 7: Succession of eukaryotic species within each bag normalized per cluster for the 2-20µm fraction. Species are clustered by similarity of their relative abundance dynamics. The lines and shaded area represent the mean and standard deviation within each cluster, respectively. Each cluster is normalized to its own maximum abundance and clusters composition is detailed **Fig. 2a**. *E. huxleyi* abundance is overlaid in black and normalized to each bag's maximum value.

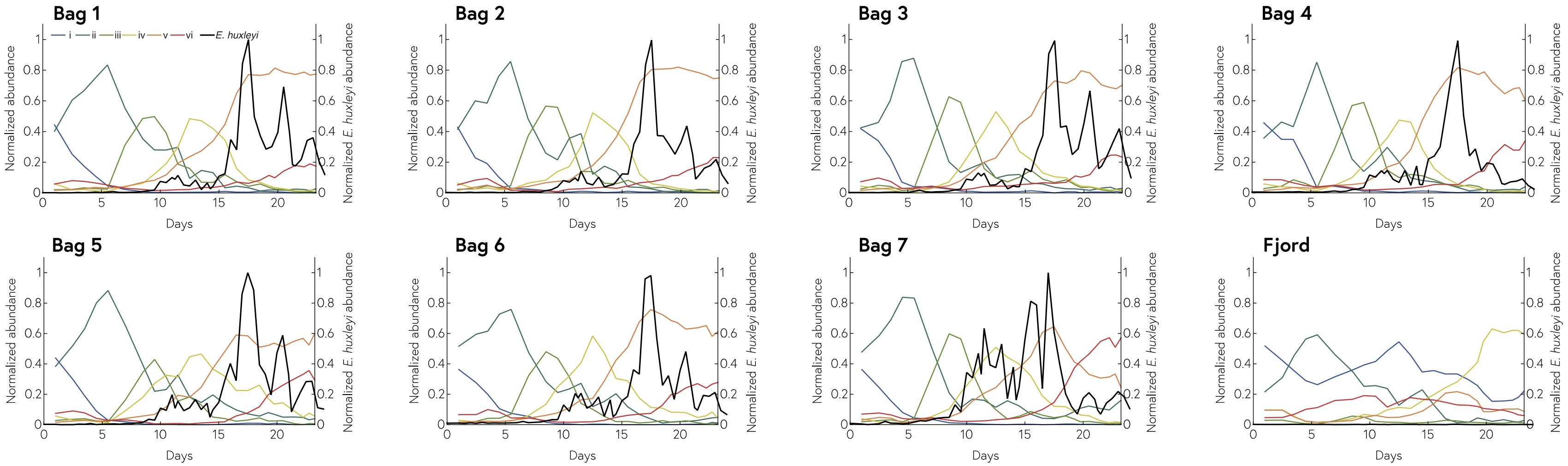

Supplementary Fig. 8: Succession of eukaryotic clusters within each bag normalized to the total relative abundance for the 2-20μm fraction. Species are clustered by similarity of their relative abundance dynamics. Each cluster is normalized to the total relative abundance and their species composition is detailed **Fig. 2a**. *E. huxleyi* abundance is overlaid in black and normalized to each bag's maximum value.

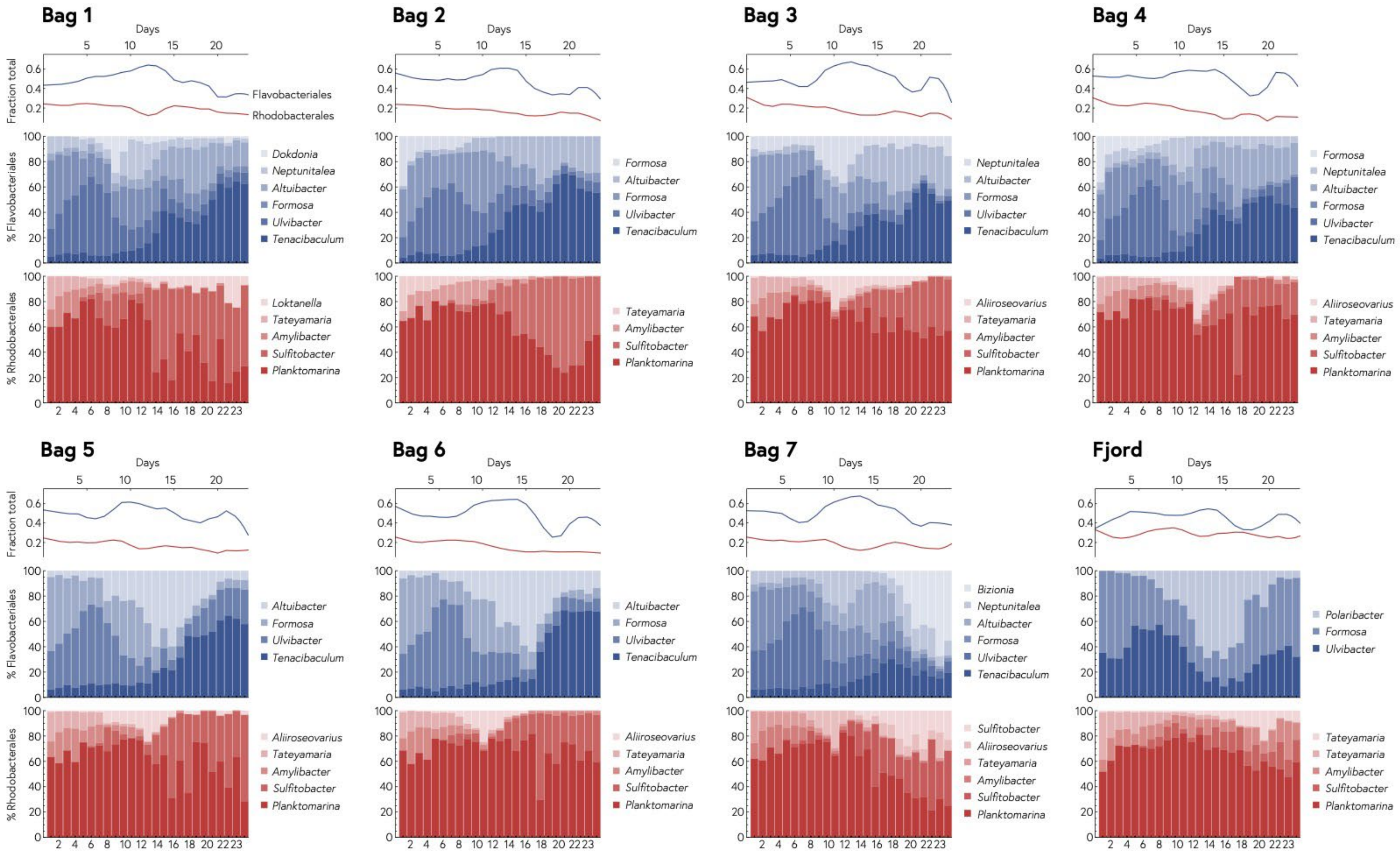

Supplementary Fig. 9: Flavobacteriales (blue) and Rhodobacterales (red) species relative abundances over time in each bag. The top pannel represents the total relative abundance of Flavobacteriales (blue) and Rhodobacterales (red) species within the whole bacterial community.

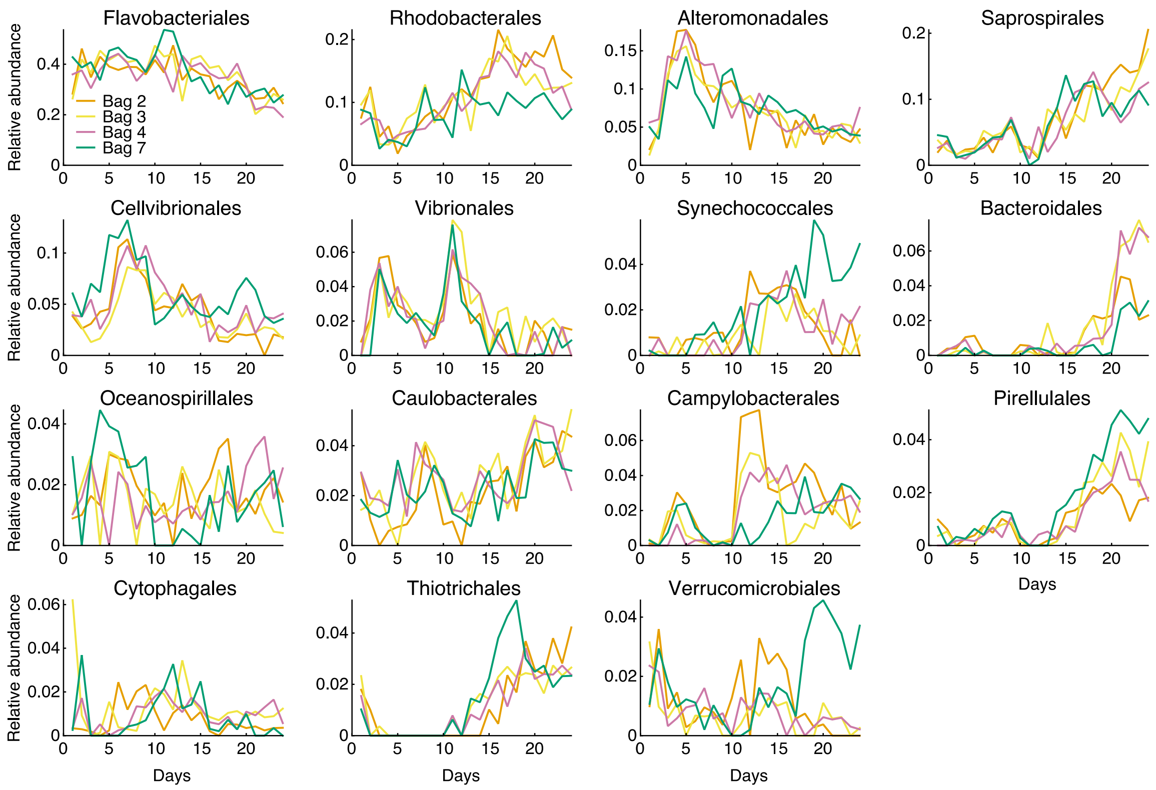

Supplementary Fig. 10: Relative abundance of major bacterial orders in the particle-associated fraction (20-200 μm) for Bags 2, 3, 4 and 7.

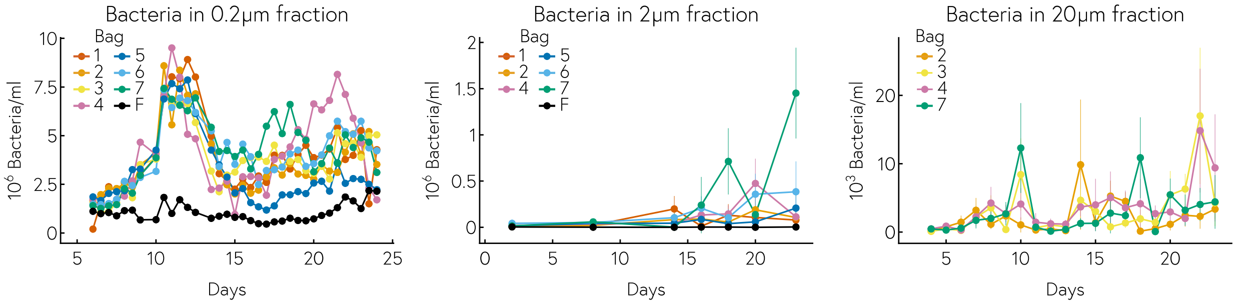

Supplementary Fig. 11: Comparison of bacterial abundances across different size fractions measured by flow-cytometry in the free-living fraction, and qPCR on filtered biomass in the larger size fractions. Points and error bars in the center and right panels represent the mean and standard deviation over three technical replicates.

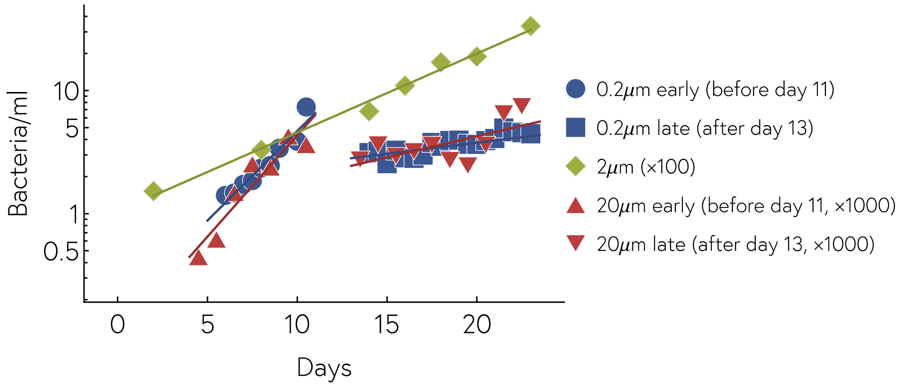

Supplementary Fig. 12: Estimated growth rate of bacteria in the different size fraction. Similarly to Fig. 3d, i.e., by fitting a line to the log-transformed absolute abundances, for the data subsets indicated by the different symbols and colors.

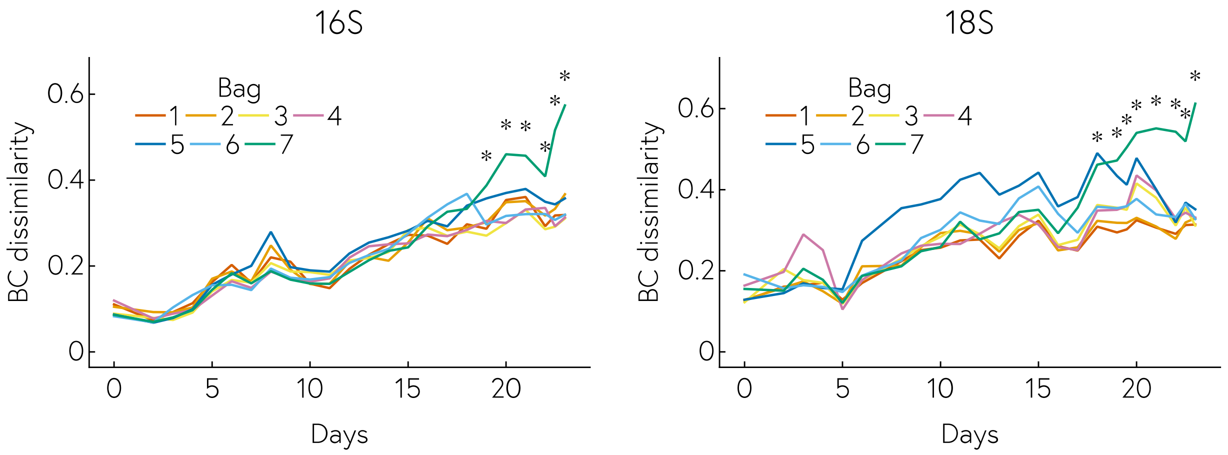

Supplementary Fig. 13: Divergence of bacterial (16S) and nanoeukaryotic (18S) compositions between bags over time. For the 18S and 16S separately, a Bray-Curtis distance between the bags was measured for each day. The stars indicate significant differences between bag 7 and the other bags according to Kolmogorov-Smirnov tests with Benjamini-Hochberg multiple testing correction at the p=0.05 level.

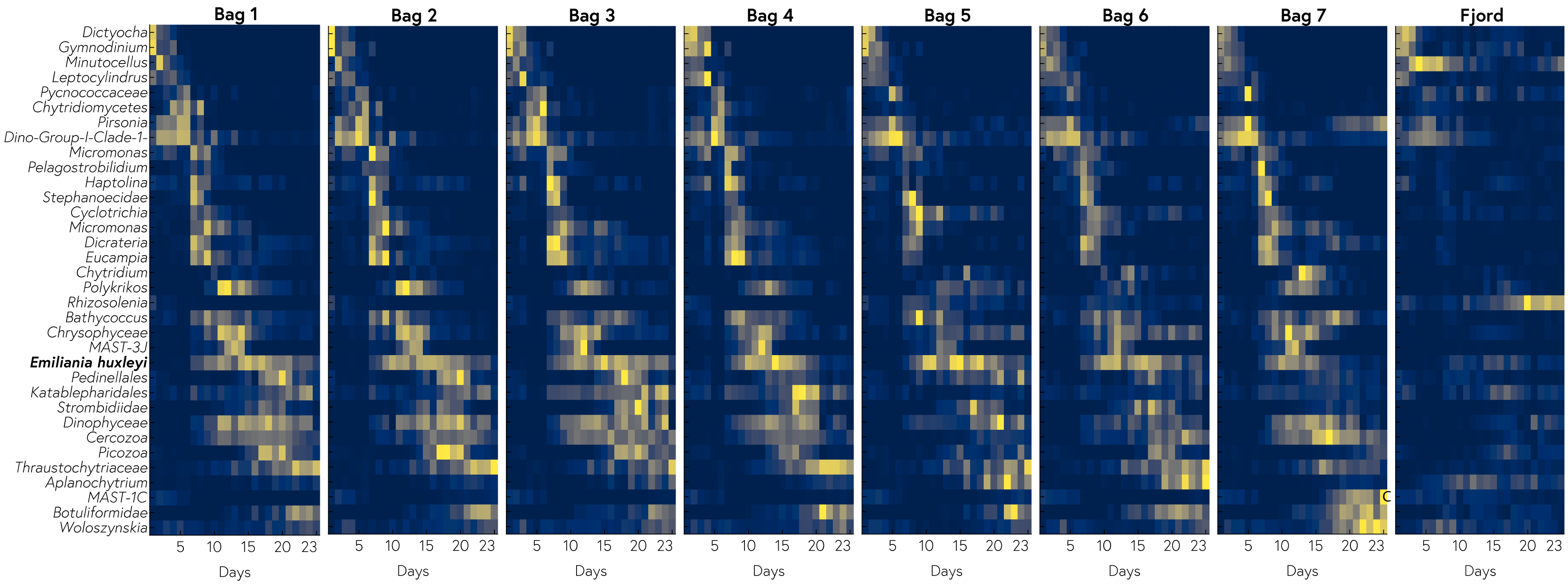

Supplementary Fig. 14: Most abundant 18S ASVs in all bags in the 2-20µm size fraction. The heatmap is normalized per row, meaning each ASV is normalized to its maximum abundance across all bags and all days.

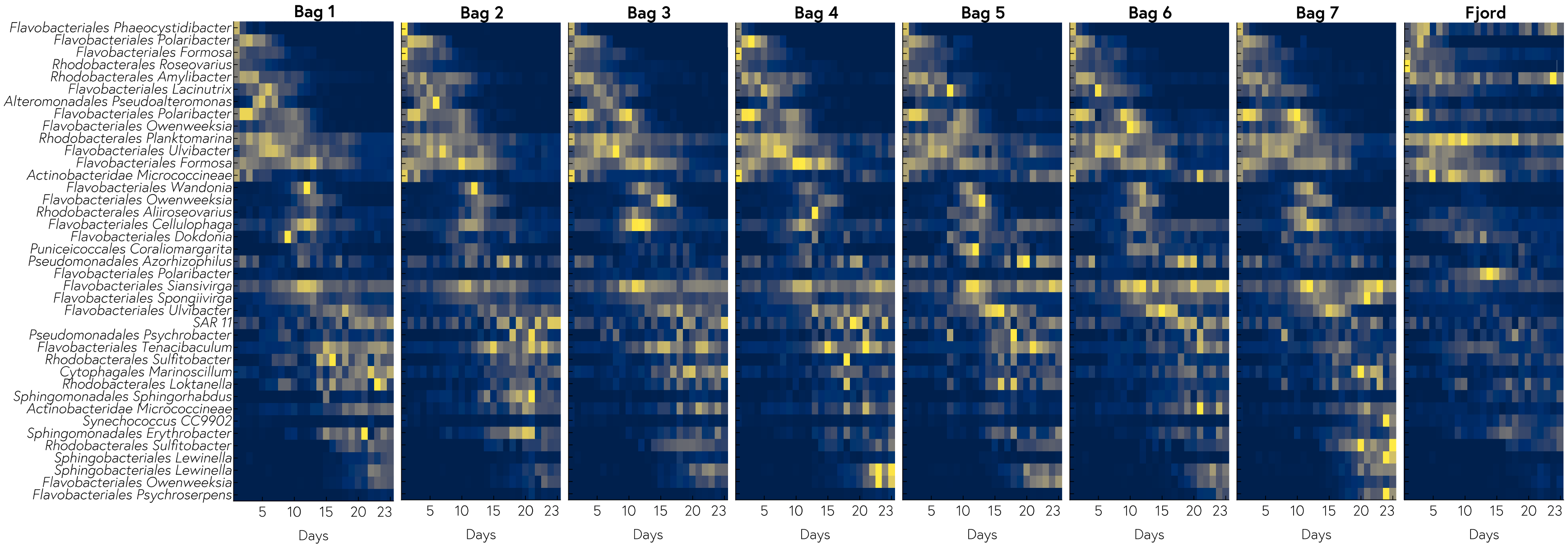

Supplementary Fig. 15: Most abundant 16S ASVs in all bags in the 0.2µm size fraction. The heatmap is normalized per row, meaning each ASV is normalized to its maximum abundance across all bags and all days.

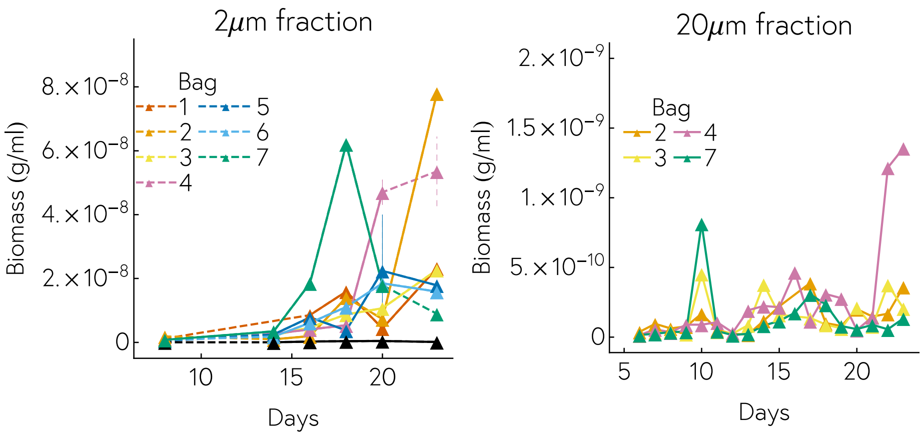

Supplementary Fig. 16: Calculated biomass in grams of carbon of thraustochytrids through time for each individual bag in the 2-20 µm (left) and 20-200 µm (right) size fractions. A conversation factor was used to transform cell abundances in cell biomass (see Supplementary Data 1, 2). Error bars indicate the standard deviation over n=2 technical replicates.

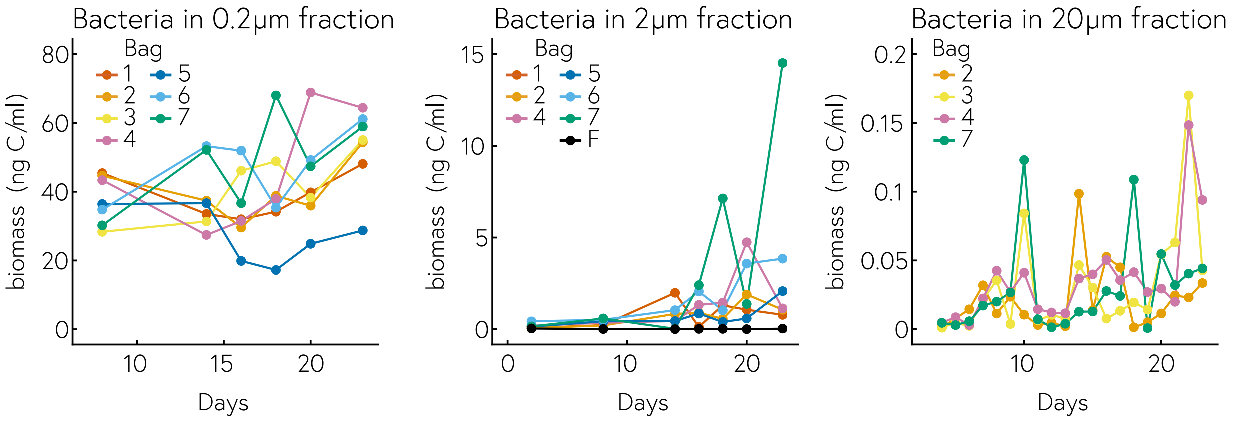

Supplementary Fig. 17: Calculated biomass of bacteria through time for each individual bag in the free-living fraction estimated by flow-cytometry (left) as well as by ddPCR in 2-20µm (middle) and 20-200µm (right) size fractions. A conversation factor was used to transform cell abundances in cell biomass (see Supplementary Data 3, 4).

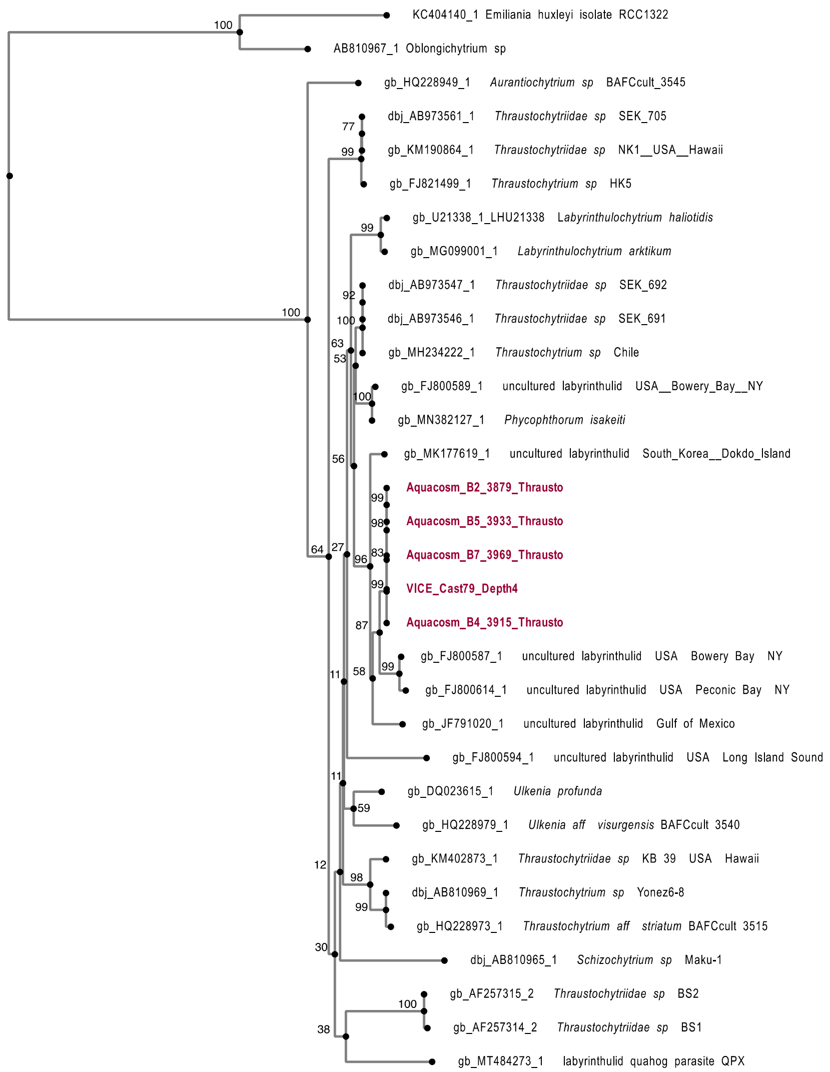

Supplementary Fig. 18: Phylogeny of thraustochytrids including new sequences from this study (in red). Aquacosm sequences all come from the Norwegian fjord. The VICE sequence comes from samples collected in the North Atlantic. The tree was based on 866 conserved sites and numeric values on the nodes were obtained with 1000 bootstraps. *E. huxleyi* and *Oblongichytrium* were used as outgroups (see Methods).

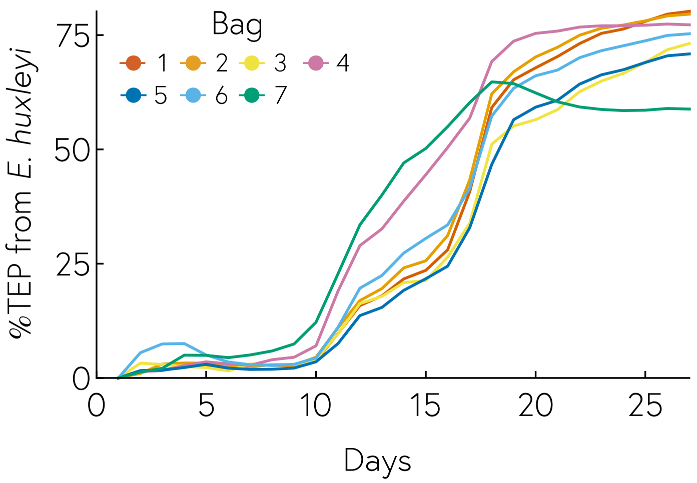

Supplementary Fig. 19: Predicted fraction of transparent exopolymers (TEP) contributed by *E. huxleyi* for each mesocosm enclosure. TEP was modelled as the sum of *E. huxleyi,* naked nanophytoplankton, and picophytoplankton TEP production, with a loss factor corresponding to TEP degradation or sinking (see Methods).

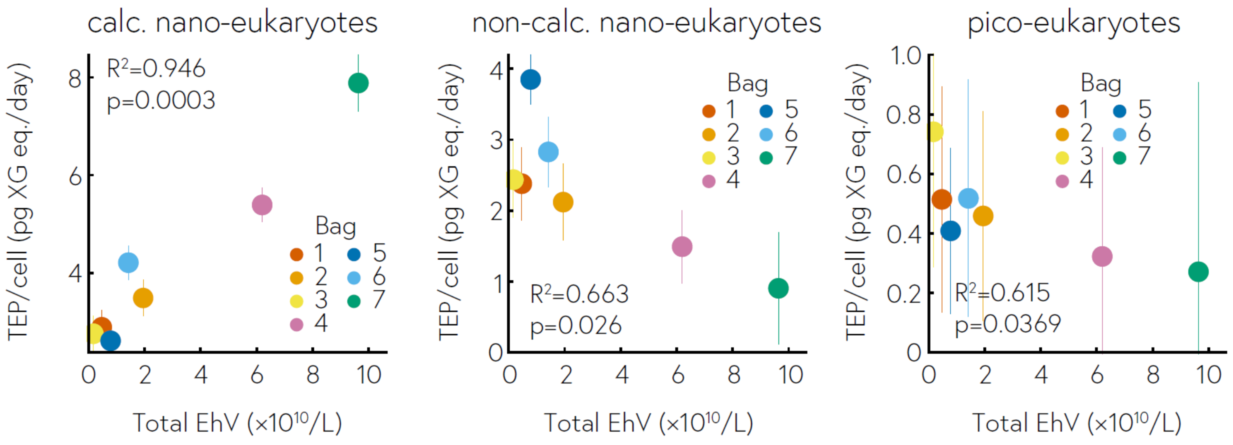

Supplementary Fig. 20: Modelled TEP production per phytoplankton population. Calcified nanophytoplankton (left) show an increase as a function of total EhV load. Non-calcified nanophytoplankton (middle), and picophytoplankton (right) cells do not show increase as a function of total EhV load. Symbols show best fit parameters, error bars represent parameter confidence intervals of the model fits.

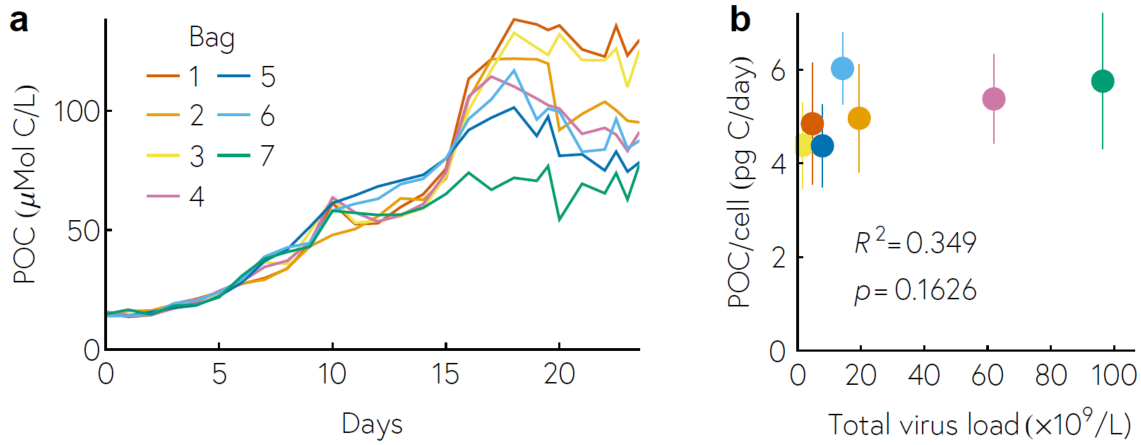

Supplementary Fig. 21: Measurements and modelling of particulate organic carbon (POC). **(A)** POC measurements over time for each individual mesocosm enclosure. **(B)** Predicted POC/cell for each individual enclosure, as a function of total viral load in each bag. The low R^2 and p values show that POC per cell is not strongly correlated with total virus load, contrary to TEP and PIC. Symbols show best fit parameters; error bars represent parameter confidence intervals of the model fits.

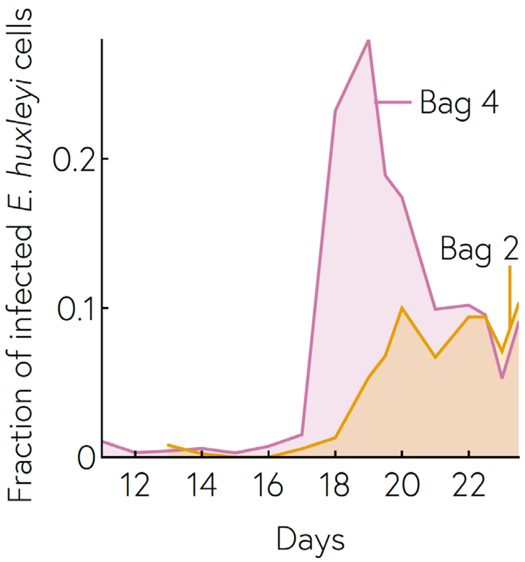

Supplementary Fig. 22: Fraction of infected *E. huxleyi* cells, measured by smFISH in bags 2 and 4. After fixation, cells were stained with a 28S probe designed to detect *E. huxleyi* cells and a probe targeting the viral *mcp* mRNA to identify actively infected cells.

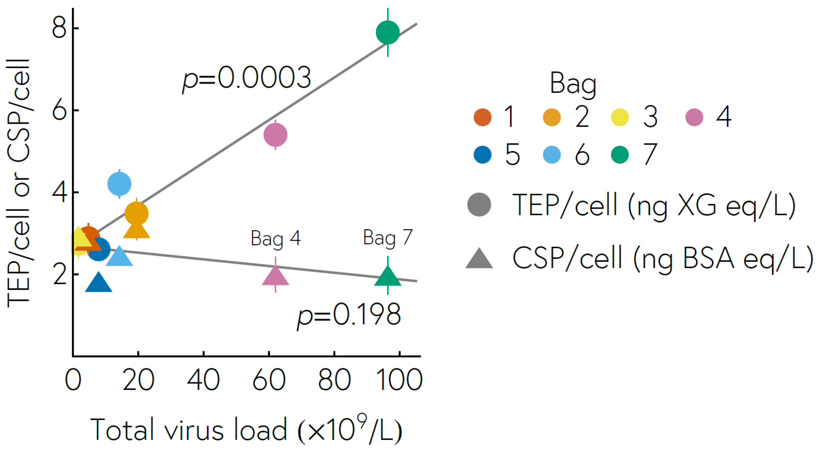

Supplementary Fig. 23: Comparison of transparent exopolymer particles (TEP) and Coomassie stainable particles (CSP) modelling. The plot displays predicted TEP and CSP per cell as a function of viral load using the same model. Non-significant fits for the CSP modelling shows that CSP is not strongly correlated with total virus load, contrary to TEP and PIC. Symbols show best fit parameters, error bars represent parameter confidence intervals of the model fits, *p* values correspond to the likelihood that the slope of the linear model fit is not significantly different from 0.

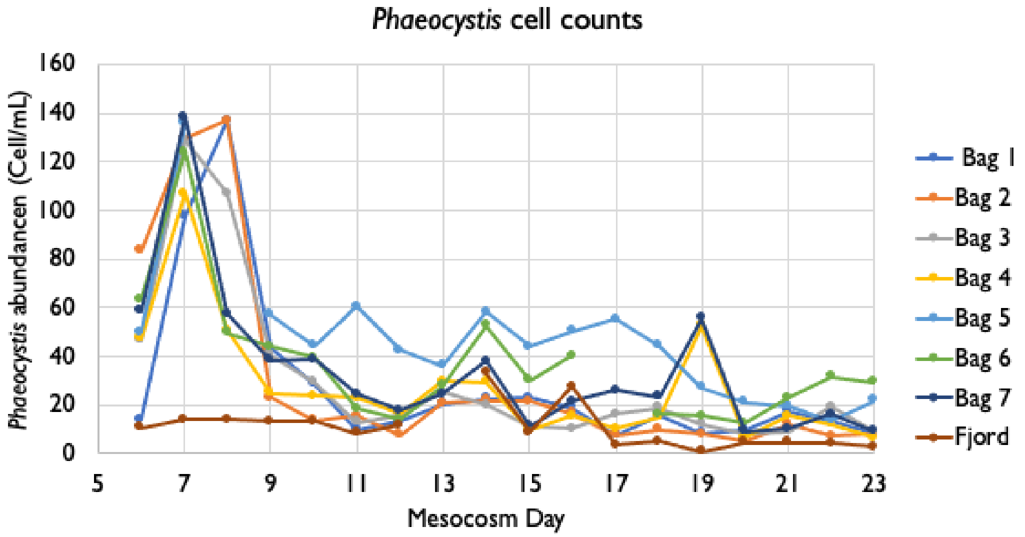

Supplementary Fig. 24: *Phaeocystis* cell counts derived from FlowCam data. To derive cell counts from *Phaeocystis* colonies, an area:cell ratio was derived from 11 colonies to obtain the following conversion factor: "cells = 0.0247*area" (R^2=0.9905).

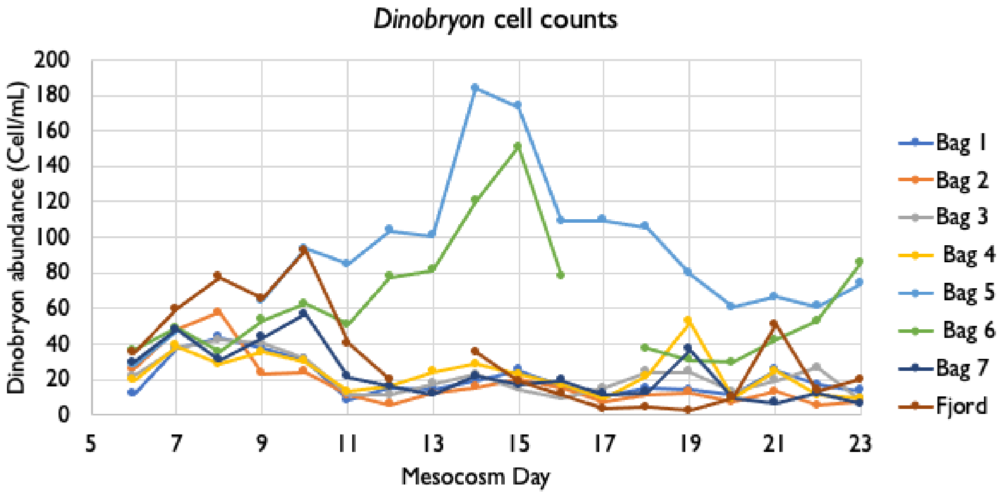

Supplementary Fig. 25: *Dinobryon* cell counts derived from FlowCam data.

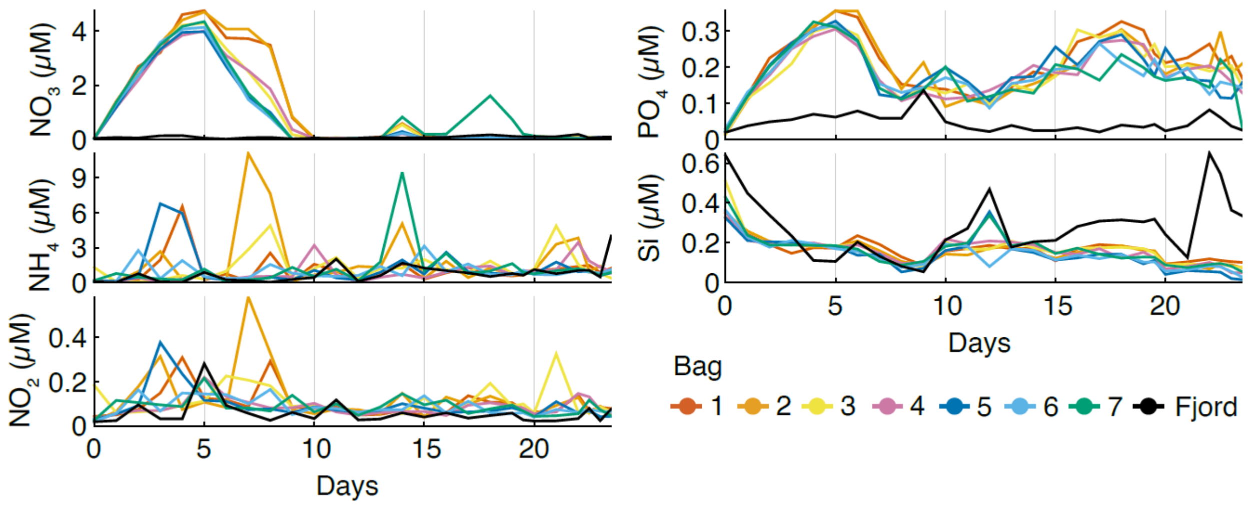

Supplementary Fig. 26: Nutrient concentrations for each individual bag across the mesocosm experiments.
