# Supplementary material for: Viral infection switches the balance between bacterial and eukaryotic recyclers of organic matter during coccolithophore blooms
Source: Nat Commun. 2023 Jan 31;14:510. doi: 10.1038/s41467-023-36049-3 (PMC9889395; doi:10.1038/s41467-023-36049-3)
Supplement: Supplementary file 2 — Description of Additional Supplementary Files [file 41467_2023_36049_MOESM2_ESM.pdf]

Description of Additional Supplementary Files

File Name: Supplementary Data 1
Description: Calculation of Thraustochytrids biomass based on ddPCR, on 2-20 um filters

File Name: Supplementary Data 2
Description: Calculation of Thraustochytrids biomass based on ddPCR, on 20-200 um filters

File Name: Supplementary Data 3
Description: Calculation of bacterial biomass based on qPCR, on 2-20 um filters

File Name: Supplementary Data 4
Description: Calculation of bacterial biomass based on qPCR, on 20-200 um filters
