# Supplementary material for: Viral infection switches the balance between bacterial and eukaryotic recyclers of organic matter during coccolithophore blooms
Source: Nat Commun. 2023 Jan 31;14:510. doi: 10.1038/s41467-023-36049-3 (PMC9889395; doi:10.1038/s41467-023-36049-3)
Supplement: Supplementary file 4 — Reporting Summary [file 41467_2023_36049_MOESM4_ESM.pdf]

nature portfolio

Corresponding author(s): Assaf Vardi

Last updated by author(s): Jan 11, 2023

Reporting Summary

Nature Portfolio wishes to improve the reproducibility of the work that we publish. This form provides structure for consistency and transparency in reporting. For further information on Nature Portfolio policies, see our Editorial Policies and the Editorial Policy Checklist.

Statistics

For all statistical analyses, confirm that the following items are present in the figure legend, table legend, main text, or Methods section.

n/a	Confirmed	
☐	☒	The exact sample size (*n*) for each experimental group/condition, given as a discrete number and unit of measurement
☐	☒	A statement on whether measurements were taken from distinct samples or whether the same sample was measured repeatedly
☐	☒	The statistical test(s) used AND whether they are one- or two-sided<br>*Only common tests should be described solely by name; describe more complex techniques in the Methods section.*
☒	☐	A description of all covariates tested
☐	☒	A description of any assumptions or corrections, such as tests of normality and adjustment for multiple comparisons
☐	☒	A full description of the statistical parameters including central tendency (e.g. means) or other basic estimates (e.g. regression coefficient) AND variation (e.g. standard deviation) or associated estimates of uncertainty (e.g. confidence intervals)
☐	☒	For null hypothesis testing, the test statistic (e.g. F, t, r) with confidence intervals, effect sizes, degrees of freedom and P value noted<br>*Give P values as exact values whenever suitable.*
☒	☐	For Bayesian analysis, information on the choice of priors and Markov chain Monte Carlo settings
☐	☒	For hierarchical and complex designs, identification of the appropriate level for tests and full reporting of outcomes
☒	☐	Estimates of effect sizes (e.g. Cohen's *d*, Pearson's *r*), indicating how they were calculated

Our web collection on statistics for biologists contains articles on many of the points above.

Software and code

Policy information about availability of computer code

Data collection	For flow cytometry: Eclipse iCyt<br>For imaging flow-cytometry: FLOWCAM II<br>For ddPCR: QX200 Biorad<br>For qPCR: QuantstudioS Thermo Fisher Scientific
Data analysis	For flow cytometry: ec800 version 1.3.7, R packages "ggcyto" and "flowcore"<br>For statistical analysis: Mathematica version 1 2.3<br>For amplicon annotation: R version 4.0.4<br>For ddPCR analysis: Quantasoft version 1.7<br>For qPCR: QuantStuodio Design and Analysis Sofware vl.5.1 For sequence alignment: mafft version 7<br>For polysaccharide analysis: Array-Pro Analyzer version 6.3

For manuscripts utilizing custom algorithms or software that are central to the research but not yet described in published literature, software must be made available to editors and reviewers. We strongly encourage code deposition in a community repository (e.g. GitHub). See the Nature Portfolio guidelines for submitting code & software for further information.

Data

Policy information about availability of data

All manuscripts must include a data availability statement. This statement should provide the following information, where applicable:

- Accession codes, unique identifiers, or web links for publicly available datasets
- A description of any restrictions on data availability
- For clinical datasets or third party data, please ensure that the statement adheres to our policy

All data needed to evaluate the conclusions in the paper are present in the paper and clearly indicated in the methods. Flow cytometry, nutrient, and temperature data are available in Dryad (https://doi.org/10.5061/dryad.q573n5tfr). Flowcam data is available on Ecotaxa under the project "Flowcam Composite Aquacosm_2018_VIMS-Ehux" (https://ecotaxa.obs-vlfr.fr/prj/2501). Sequencing data has been deposited under NCBI Bioproject PRJNA694552: 16S data is available under Biosample SAMN17576248 and 18S data is available under Biosample SAMN20295136. Assembled sequences deposited on NCBI with accession numbers MZ562737, MZ562738, MZ562739, MZ562740, MZ562741.

Human research participants

Policy information about studies involving human research participants and Sex and Gender in Research.

Reporting on sex and gender	*Use the terms sex (biological attribute) and gender (shaped by social and cultural circumstances) carefully in order to avoid confusing both terms. Indicate if findings apply to only one sex or gender; describe whether sex and gender were considered in study design whether sex and/or gender was determined based on self-reporting or assigned and methods used. Provide in the source data disaggregated sex and gender data where this information has been collected, and consent has been obtained for sharing of individual-level data; provide overall numbers in this Reporting Summary. Please state if this information has not been collected. Report sex- and gender-based analyses where performed, justify reasons for lack of sex- and gender-based analysis.*
Population characteristics	*Describe the covariate-relevant population characteristics of the human research participants (e.g. age, genotypic information, past and current diagnosis and treatment categories). If you filled out the behavioural & social sciences study design questions and have nothing to add here, write "See above."*
Recruitment	*Describe how participants were recruited. Outline any potential self-selection bias or other biases that may be present and how these are likely to impact results.*
Ethics oversight	*Identify the organization(s) that approved the study protocol.*

Note that full information on the approval of the study protocol must also be provided in the manuscript.

Field-specific reporting

Please select the one below that is the best fit for your research. If you are not sure, read the appropriate sections before making your selection.

☐ Life sciences ☐ Behavioural & social sciences ☒ Ecological, evolutionary & environmental sciences

For a reference copy of the document with all sections, see nature.com/documents/nr-reporting-summary-flat.pdf

Ecological, evolutionary & environmental sciences study design

All studies must disclose on these points even when the disclosure is negative.

Study description	This study analyses a month-long time series of natural marine microbial communities across seven large mesocosm bags of 11,000L each. Daily sampling was performed, to characterize the bacterial and eukaryotic microbiomes, as well as biogeochemical dynamics such as polysaccharides and inorganic carbon.
Research sample	The sample are seven big mesocosm bags filled with natural fjord water on the first day of the experiment.<br>Each bag has been supplemented with nutrients to trigger a phytoplankton bloom.<br>Water from the surrounding fjord has been sampled daily, and used as a negative control.
Sampling strategy	The choice of using seven mesocosm bags corresponds to diverse contraints. First, the size of the raft does not enable more than 7 mesocosm bags to be attached.<br>Then, three of our mesocosm had an air-tight cover to enable the sampling of aerosols. Three bags were chosen to have statistical power.<br>The remaining four bags were therefore uncovered.
Data collection	This study is part of a large consortium, including local (Norwegian) partners, and international labs.<br>Every team was responsible for a different aspect of this global study, chosen according to the respective lab expertise.

	The VARDI Lab lead the study and was responsible for a substantial part of the data collection and processing to date (flow cytometry, imaging flow cytometry, biomass filters). The Simo Lab collected all dataset related to biogeochemistry. The Heheman Lab collected data related to polysaccharide analysis. The Cordero Lab collected prokaryotic biomass.
Timing and spatial scale	The mesocosm bags were installed and filled with natural sea water on 23/05/2021.<br>Samples were taken once a day in the morning, until 16/06/2021.<br>On the 12/06, 15/06 and 16/06, samples were taken twice a day, both in the morning and in the evening.
Data exclusions	Evening samples from 12/06/18, 15/06/18 and 16/06/18 were excluded from this study as they were specifically collected for a dial cycle experiment not included in this paper.
Reproducibility	The mesocosm experiment is a field experiment, and therefore by definition cannot be strictly reproduced.<br>When applicable, several replicates were performed to acquire the data (qPCR for viral counts, flow cytometry)
Randomization	On the first day, instead of filling each bag completely one by one, the seven bags were gradually filled up to maximize homogeneity across bags and thus random distribution of microbial species across the seven mesocosm enclosures.
Blinding	Blinding is not relevant for this study as the data acquisition was done in the natural environment with clearly identified bags.

Did the study involve field work? ☒ Yes ☐ No

Field work, collection and transport

Field conditions	All the data regarding field conditions has been made available on Dryad doi.org/10.5061/dryad.q573n5tfr
Location	The mesocosm experiment AQUACOSM VIMS-Ehux was carried out for 24 days between 23rd May (day 0) and 16th June (day 23) in 2018 in Raunefjorden at the University of Bergen's Marine Biological Station Espegrend, Norway (60°16"11N; 5°13"07E).
Access & import/export	All efforts to access, import, export and use samples or scientific equipments has been conducted in coordination with local scientific partners in absolute compliance with local regulation along with Nagoya Protocols.
Disturbance	The disturbance cause is that at the end of the mesocosm experiement, the content of the bags is released in the natural environment. The disturbance is minimized by the low volumes remaining (after 23 days sampling) and does not introduce any foreign agent in the water as everything in the bags was initiated from a natural community

Reporting for specific materials, systems and methods

We require information from authors about some types of materials, experimental systems and methods used in many studies. Here, indicate whether each material, system or method listed is relevant to your study. If you are not sure if a list item applies to your research, read the appropriate section before selecting a response.

Materials & experimental systems

n/a	Involved in the study
☐	☒ Antibodies
☒	☐ Eukaryotic cell lines
☒	☐ Palaeontology and archaeology
☐	☒ Animals and other organisms
☒	☐ Clinical data
☒	☐ Dual use research of concern

Methods

n/a	Involved in the study
☒	☐ ChIP-seq
☐	☒ Flow cytometry
☒	☐ MRI-based neuroimaging

Antibodies

Antibodies used	BAM1,BAM2, BAM7,BAM6, the monoclonal antibodies used in this study, were raised in rats and developed by scientists in the laboratory of Prof. Paul Knox at the University of Leeds UK and supervised by Dr. Cecile Herve, CNRS researcher at the Station Biologique de Roscoff in France. The BAM1,BAM2, BAM7,BAM6 antibodies can be obtained from Dr. Cecile Herve through the homepage: https://www.sb-roscoff.fr/en/seaprobes
Validation	The validation of these antibodies was carried out by Dr. Thomas Torode et al. (2015 PlosOne) while working in the laboratory of Prof. Paul Knox with Dr. Cecile Herve. BAM1 recognizes un-sulfated epitope present in sulfated fucan/fucoidan preparations and BAM2 recognizes sulfated epitope present in sulfated fucan/fucoidan preparations. BAM6 recognizes mannuronate-rich epitope and BAM7 recognizes mannuronate-guluronate. The detailed specifications and validations of these antibodies have been described in this Plos One article: https://doi.org/10.1371/journal.pone.0118366

The ability of BAM1 and BAM2 to detect sulfated fucose containing polysaccharides (FCSPs) in microalgal blooms was previously verified in this publication https://doi.org/10.1038/s41467-021-21009-6

Animals and other research organisms

Policy information about studies involving animals; ARRIVE guidelines recommended for reporting animal research, and Sex and Gender in Research

Laboratory animals	N/A
Wild animals	The only wild animals in this study are small crustaceans, in very low concentration, that have been filtered and sequenced.
Reporting on sex	*Indicate if findings apply to only one sex; describe whether sex was considered in study design, methods used for assigning sex. Provide data disaggregated for sex where this information has been collected in the source data as appropriate; provide overall numbers in this Reporting Summary. Please state if this information has not been collected. Report sex-based analyses where performed, justify reasons for lack of sex-based analysis.*
Field-collected samples	All efforts to access, import, export and use samples or scientific equipments has been conducted in coordination with local scientific partners in absolute compliance with local regulation along with Nagoya Protocols.
Ethics oversight	No organization has provided specific advice ethical advise regarding marine microbial research conducted in this study.

Note that full information on the approval of the study protocol must also be provided in the manuscript.

Flow Cytometry

Plots

Confirm that:

- [x] The axis labels state the marker and fluorochrome used (e.g. CD4-FITC).
- [x] The axis scales are clearly visible. Include numbers along axes only for bottom left plot of group (a 'group' is an analysis of identical markers).
- [x] All plots are contour plots with outliers or pseudocolor plots.
- [ ] A numerical value for number of cells or percentage (with statistics) is provided.

Methodology

Sample preparation	Water samples were collected in 50 ml centrifugal tubes from 1 m depth, pre-filtered using 40 µm cell strainers.
Instrument	Samples were immediately analyzed with an Eclipse iCyt (Sony Biotechology, Champaign, IL, USA) flow cytometer.
Software	ec800 version 1.3.7
Cell population abundance	Cell population abundance was considered when containing only above 100 absolute events.
Gating strategy	Phytoplankton populations were identified by plotting the autofluorescence of chlorophyll versus phycoerythrin and side scatter: calcified E. huxleyi (high side scatter and high chlorophyll), Synechococcus (high phycoerythrin and low chlorophyll), nano- and picophytoplankton (high and low chlorophyll, respectively). Chlorophyll fluorescence was detected by FL4 (excitation (ex): 488nm and emission (em): 663-737 nm). Phycoerythrin was detected by FL3 (ex: 488 nm and em: 570-620 nm). All the gates are visible in the supplementary information link https://datadryad.org/stash/dataset/doi:1.561/dryad.q573n5tfr, specifically "All .pdf documents containing the suffix "_Gates" represent the different phytoplankton population gates per bag."<br>This pdf file contains, for every day and every bag, a plot with the gating strategy.

- [x] Tick this box to confirm that a figure exemplifying the gating strategy is provided in the Supplementary Information.
